# Wastewater early warning system for SARS-CoV-2 outbreaks and variants in a Coruña, Spain

**DOI:** 10.1007/s11356-023-27877-3

**Published:** 2023-06-07

**Authors:** Noelia Trigo-Tasende, Juan A. Vallejo, Soraya Rumbo-Feal, Kelly Conde-Pérez, Manuel Vaamonde, Ángel López-Oriona, Inés Barbeito, Mohammed Nasser-Ali, Rubén Reif, Bruno K. Rodiño-Janeiro, Elisa Fernández-Álvarez, Iago Iglesias-Corrás, Borja Freire, Javier Tarrío-Saavedra, Laura Tomás, Pilar Gallego-García, David Posada, Germán Bou, Ignacio López-de-Ullibarri, Ricardo Cao, Susana Ladra, Margarita Poza

**Affiliations:** 1 University of A Coruña (UDC) - Microbiome and Health group (meiGAbiome), Institute of Biomedical Research (INIBIC) - University Hospital of A Coruña (CHUAC) - Interdisciplinary Center for Chemistry and Biology (CICA) - Spanish Network for Infectious Diseases (CIBERINFEC-ISCIII), Campus da Zapateira, 15008 A Coruña, Spain; 2 Research Group MODES, Research Center for Information and Communication Technologies (CITIC), University of A Coruña (UDC), Campus de Elviña, 15071 A Coruña, Spain; 3 Center for Research in Biological Chemistry and Molecular Materials (CiQUS), University of Santiago de Compostela (USC), 15782 Santiago de Compostela, Spain; 4 BFlow, University of Santiago de Compostela (USC) and Health Research Institute of Santiago de Compostela (IDIS), Campus Vida, 15706 Santiago de Compostela, A Coruña, Spain; 5 University of A Coruña (UDC), Research Center for Information and Communication Technologies (CITIC), Database Laboratory, Campus de Elviña, 15071 A Coruña, Spain; 6 CINBIO, Universidade de Vigo, 36310 Vigo, Spain; 7 Galicia Sur Health Research Institute (IIS Galicia Sur), SERGAS-UVIGO, 36312 Vigo, Spain; 8 Department of Biochemistry, Genetics, and Immunology, Universidade de Vigo, 36310 Vigo, Spain

**Keywords:** SARS-CoV-2, Statistical model, Wastewater-based epidemiology, Early-warning system, Viral load, COVID-19

## Abstract

Wastewater-based epidemiology has been widely used as a cost-effective method for tracking the COVID-19 pandemic at the community level. Here we describe COVIDBENS, a wastewater surveillance program running from June 2020 to March 2022 in the wastewater treatment plant of Bens in A Coruña (Spain). The main goal of this work was to provide an effective early warning tool based in wastewater epidemiology to help in decision-making at both the social and public health levels. RT-qPCR procedures and Illumina sequencing were used to weekly monitor the viral load and to detect SARS-CoV-2 mutations in wastewater, respectively. In addition, own statistical models were applied to estimate the real number of infected people and the frequency of each emerging variant circulating in the community, which considerable improved the surveillance strategy. Our analysis detected 6 viral load waves in A Coruña with concentrations between 10^3^ and 10^6^ SARS-CoV-2 RNA copies/L. Our system was able to anticipate community outbreaks during the pandemic with 8–36 days in advance with respect to clinical reports and, to detect the emergence of new SARS-CoV-2 variants in A Coruña such as Alpha (B.1.1.7), Delta (B.1.617.2), and Omicron (B.1.1.529 and BA.2) in wastewater with 42, 30, and 27 days, respectively, before the health system did. Data generated here helped local authorities and health managers to give a faster and more efficient response to the pandemic situation, and also allowed important industrial companies to adapt their production to each situation. The wastewater-based epidemiology program developed in our metropolitan area of A Coruña (Spain) during the SARS-CoV-2 pandemic served as a powerful early warning system combining statistical models with mutations and viral load monitoring in wastewater over time.

**Supplementary Information:**

The online version contains supplementary material available at 10.1007/s11356-023-27877-3.

## Introduction

SARS-CoV-2, the etiological agent responsible for the deadly respiratory disease known as COVID-19, emerged in Wuhan (China) in early December 2019. It was on March 11, 2020, when the World Health Organization (WHO) declared the novel coronavirus outbreak a global pandemic (WHO 2020). The transmission dynamics of COVID-19 is due to two mechanisms: human-to-human and air pollution-to-person transmission. The first one is through respiratory droplets and direct contact with infected people, and it is influenced by the population density. However, Coccia (2020) highlighted the importance of the air pollution in the rapid spread of COVID-19. They suggested that climate and meteorology factors such as pollution or wind which affect the content of microbes in the atmosphere, accelerating the spread of SARS-CoV-2 in the environment, including wastewater. Besides, although the fecal–oral transmission of SARS-CoV-2 has not been proved yet (Albert et al. 2021; Guo et al. 2021), COVID-19 dissemination could increase due to inadequate sanitation facilities or poor fecal sludge management in hospitals environments (Amin et al. 2023).

The viral surface spike glycoprotein S plays an important role in the infection process, mediating viral entry into host cells through the union of the spike receptor-binding domain (RBD) and the angiotensin-converting enzyme2 (ACE2) receptor (Lan et al. 2020). ACE2 is abundant in glandular cells of the gastric, duodenal, and rectal epithelium (Xiao et al. 2020), where the virus persists longer than in the respiratory tract (Hu et al. 2020; Zheng et al. 2020). Therefore, people infected with SARS-CoV-2 shed the virus in their feces despite being asymptomatic (Lai et al. 2020; Rothe et al. 2020) or having tested negative in nasopharyngeal samples (Jiang et al. 2020; Wu et al. 2020). It has been shown that the virus can remain in stool samples for up to 5 weeks after the onset of symptoms (Wu et al. 2020). As a result, SARS-CoV-2 RNA from most infected patients ends up in wastewater treatment plants (WWTPs). Therefore, the analysis of wastewater samples can provide effective epidemiological surveillance.

Wastewater-based epidemiology (WBE) has been used as a method of early detection and direct mitigation of poliovirus outbreaks in Israel and Egypt (Blomqvist et al. 2012; Brouwer et al. 2018; Kopel et al. 2014) or Norovirus and Hepatitis A in Sweden (Berchenko et al. 2017; Duintjer Tebbens et al. 2017; Hellmér et al. 2014). Currently, RT-qPCR assays are used for clinical trials and the detection of viral RNA in WWT (Acosta et al. 2022; Kolarević et al. 2022; Tanimoto et al. 2022; Yanaç et al. 2022).

In most WBE SARS-CoV-2 studies, a significant correlation has been found between the viral load measured in wastewater and clinical cases of COVID-19 (Maida et al. 2022; Pillay et al. 2021; Vallejo et al. 2022), which demonstrates that an increase on viral load in WWT can warn about the emergence of a new variant or an outbreak (Barua et al. 2022; Daleiden et al. 2022; Kuhn et al. 2022; Li et al. 2022; Monteiro et al. 2022; Padilla-Reyes et al. 2022; Robotto et al. 2022; Sangsanont et al. 2022; Wu et al. 2022). In addition, WBE has been used to estimate the number of people infected with SARS-CoV-2 in the population (Chavarria-Miro et al. 2021; McMahan et al. 2021; Saththasivam et al. 2021; Tharak et al. 2022; Vallejo et al. 2022). However, there are few studies focused on monitoring the virus and predicting COVID-19 clinical cases in specifically rural areas or at the building level (Jarvie et al. 2023). It should be noted that the effectiveness of the post-factum methods in WBE studies, that is, an early warning analysis based on previously reported both wastewater and clinical data, has been proved (Zhao et al. 2023). The implementation of both post-factum and real-time early warning methods in a parallel and complementary way could increase the precision of the WBE analysis by almost 100%.

The emergence of new SARS-CoV-2 variants since late 2020 prompted the classification by the European Centre for Disease Prevention and Control (ECDC) of Variants of Interest (VOI) Variants of Concern (VOC), Variants Under Monitoring (VUM), and de-escalated variants (ECDC 2020). VOCs have a significant epidemiological impact due to their increase in transmissibility or in the severity of the disease they cause (Gobeil et al. 2021; Harvey et al. 2021; Riou et al. 2022). From the beginning of the pandemic up to now, Alpha (B.1.1.7), Beta (B.1.351), Gamma (P.1), Delta (B.1.617.2), and Omicron (B.1.1.529) VOCs have been most relevant causing different epidemic waves. The emergence of the variants has made the need for sequencing in wastewater more evident. On March 17, 2021, the European Commission (EC) published several recommendations to foster the surveillance of SARS-CoV-2 and its variants in wastewater (EC 2021a). Not surprisingly, genomic surveillance and monitoring are highly effective in detecting the emergence of SARS-CoV-2 variants in imported cases and in small outbreaks (Fontenele et al. 2021; Pechlivanis et al. 2022; Pérez-Cataluña et al. 2022; Peterson et al. 2022; Sapoval et al. 2021). Moreover, our team has been previously focused on estimating the proportions of variants in the population based on mutations data found in wastewater samples using statistical models (López de Ullibarri et al. 2023). Similar models have also been described by other authors (Gafurov et al. 2022; Pipes et al. 2022; Valieris et al. 2022).

The COVIDBENS project was one of the earliest WBE on COVID-19 projects in the world, starting on April 14, 2020 (Vallejo et al. 2022). In the present work, we analysed 863 wastewater samples from June 2020 to March 2022 collected from the WWTP Bens (A Coruña, Spain), a public company that serves a population of ca. 400.000 inhabitants. The main objectives of this multidisciplinary study were the following: (1) to monitor at real time the COVID-19 pandemic by tracking SARS-CoV-2 viral load in wastewater in the metropolitan area of A Coruña; (2) to estimate the number of people infected with SARS-CoV-2 in the local population, including symptomatic and asymptomatic people, using statistical models described before by our team (Vallejo et al. 2022); (3) to act as an early warning system for predicting new outbreaks before the health system; (4) to detect SARS-CoV-2 mutations in wastewater by next-generation sequencing (NGS); (5) to estimate the percentage of SARS-CoV-2 variants based on the mutations found in the wastewater using previous statistical models described by our team (López de Ullibarri et al. 2023); and (6) to inform the authorities and all citizens about the real evolution of the COVID-19 pandemic providing a public service. In this manuscript, we describe how COVIDBENS alerted about the increase of SARS-CoV-2 in the population on multiple occasions, predicting the most significant outbreaks during the COVID-19 pandemic and the emergence of the different variants that affected this area. COVIDBENS has been extremely useful as an early warning system for decision-making and for evaluating the impact of SARS-CoV-2 control measures or vaccination campaigns in our local area, A Coruña (Spain).

## Materials and methods

### Sample and data

#### Wastewater sampling

**Fig. 1:**
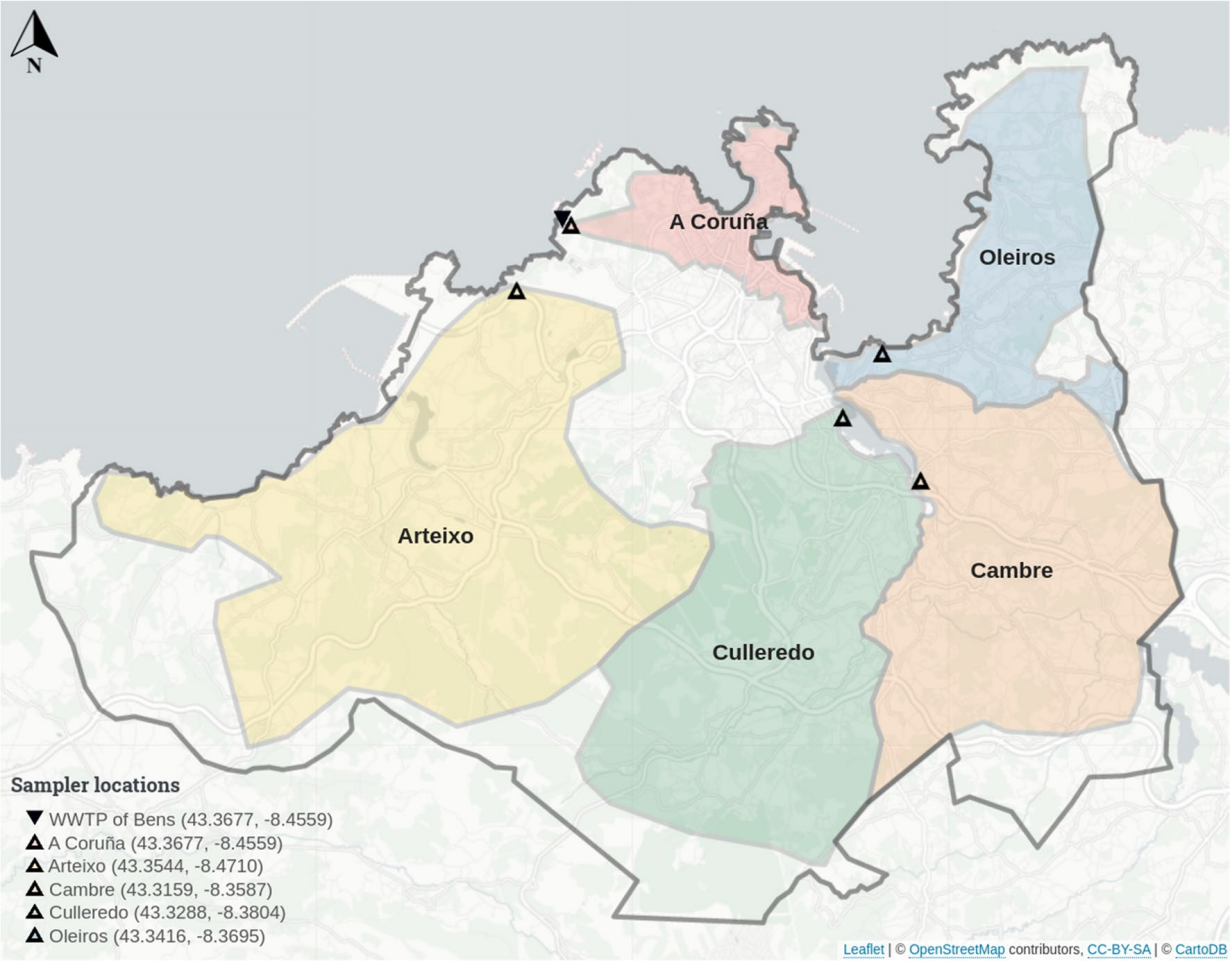
Map showing the sampling area served by the WWTP of Bens (▲), including the areas of the municipalities of A Coruña, Arteixo, Cambre, Oleiros, and Culleredo which correspond to individual sewers

The WWTP of Bens serves a population of ca. 400.000 inhabitants from the metropolitan area of A Coruña (NW, Spain) that includes the municipalities of A Coruña, Arteixo, Cambre, Culleredo, and Oleiros (Fig. 1). Composite sewage samples were collected twice or thrice a week using automatic samplers installed by operators both at the entrance (influent) of the WWTP and at the sewers of each municipality. These automatic samplers were programmed to collect 150 mL of wastewater every 15 min over a 24-h period, resulting in a 600 mL bottle per hour. Then, the resulting 24 bottles were merged into a larger one, and a sample of 100 mL was kept on ice and processed immediately after reception at the lab*.* For the present study, samples from the WWTP were collected from June 4, 2020, to March 17, 2022, and, in the case of A Coruña, Arteixo, Cambre, Culleredo, and Oleiros, were collected separately from December 24, 2020, to March 17, 2022.

#### Sample processing

The 100 mL samples were centrifuged for 30 min at 4000 × *g* at 4 °C and filtered through 0.22-μm pore membranes (Merck Millipore, USA). Pellets were discarded, and the supernatants were concentrated and dialyzed with SM buffer (50 mM Tris–HCL, 100 mM NaCl, and 8 mM MgSO4) using the Vivaspin Turbo 15 with a polyethersulfone membrane of 30-kDa (Sartorius, Germany). Finally, samples were preserved in 500 μL of RNAlater™ solution (Thermo Fisher Scientific, USA) at − 80 °C for further analyses.

For viral load determination, SARS-CoV-2 RNA was isolated from 100 μL of the thawed samples using the QIAamp Viral RNA Mini Kit (Qiagen, Germany), according to the manufacturer’s protocol. Samples were first mixed with AVL buffer supplemented with carrier RNA to ensure the binding of viral RNA to the QIAamp silica-based membrane**.** Then, samples were washed twice to remove PCR inhibitors and eluted in 70 μL of RNase-free water (Thermo Fisher Scientific, USA). For sequencing, RNA was extracted again from the remaining amount of sample (400 μL) and eluted in 80 μL of RNase-free water (Thermo Fisher Scientific, USA). Extracted RNA was stored at − 80 °C until use.

SARS-CoV-2 viral RNA was detected by one-step real-time reverse transcription quantitative PCR (RT-qPCR) using the TaqPath COVID-19 RT-PCR Kit (Thermo Fisher Scientific, USA) and a CFX96 Thermal cycler (Bio-Rad, USA). This kit includes primer pairs targeting SARS-CoV-2 specific genome regions such as ORF1ab, the N gene, and the S gene. Recommendations for minimising errors in RT–PCR detection were followed (Ahmed et al. 2022). Reactions were initiated with an uracil N-glycosylase (UNG) incubation at 25 °C for 2 min and a reverse transcription at 53 °C for 10 min, followed by a polymerase activation at 95 °C for 2 min; 40 cycles of 95 °C for 3 s for DNA denaturation; and 60 °C for 30 s for annealing and extension. A 25 μL reaction contained 15 μL of reaction mix (6.25 μL of TaqPath 1-Step Multiplex Master Mix, 1.25 μL of COVID-19 Real-Time PCR Assay Multiplex, and 7.5 μL of nuclease-free water) and 10 μL of viral RNA extracted from wastewater samples. All the quantitative assays were performed in sextuplicate. All the RT-qPCR reactions were performed using Hard-Shell 96-well PCR plates (Bio-Rad, USA) sealed with microseal “B” PCR Plate Sealing Film (Bio-Rad, USA). A non-template control (NTC) was included on each plate by replacing RNA with 10 μL of RNase-free water (Thermo Fisher Scientific, USA). Positive controls provided in the kit were also included for quality control.

#### Genomic library preparation and sequencing

Samples from the WWTP and from the municipality of A Coruña with lower Cq values were selected weekly for genomic library construction. cDNA synthesis was prepared from 11 μL of RNA using the SuperScript IV Reverse Transcriptase (Thermo Fisher Scientific, USA) and then amplified with ARTIC primer set v3 (Artic-network 2020). The resulting amplicons were mixed and purified with AMPure XP magnetic beads (Beckman Coulter, USA), and concentration was quantified with Qubit dsDNA HS Assay kit (Invitrogen, USA), attending manufacturer’s instructions. A total of 100 ng of DNA were taken into library preparation using the Illumina DNA Prep kit (Illumina, USA). Library concentration was measured using the Qubit dsDNA HS assay kit (Invitrogen, USA), and library validation and mean fragment size was determined using a Bioanalyzer DNA Analysis Kit (Agilent Technologies, USA). After dilution to 4 nM, libraries were pooled, denatured, diluted to 10 pM, and finally sequenced at read length 2 × 150 bp using the MiSeq Reagent Kit v2 (300 cycles) (Illumina, USA) and a MiSeq platform (Illumina, USA).

### Measures of variables

Viral load was determined using the human 2019-nCoV RNA standard from European Virus Archive Global (EVAg). Calibration standards were prepared by diluting a stock solution of EVAg (10,000 copies/μL) in RNase-free water (Thermo Fisher Scientific, USA) within the range of 5–500 copies/μL for SARS-CoV-2. For each assay, a linear regression fit was established between log10 SARS-CoV-2 copy number and the Cq (quantification cycle) values for the N gene. A linear fit (*y* = *mx* + *b*) was obtained where *y* is SARS-CoV-2 RNA copies per L, *x* is the RT-qPCR Cq value, *m* is the slope, and *b is* the y-intercept. The calibration was included in every RT-qPCR run. Viral load in wastewater samples was calculated by using the slope and the *y*-intercept of the corresponding standard curves and the Cq values from RT-qPCR data.

The limit of detection (LoD) and the limit of quantification (LoQ) were determined from 20 replicates of the standard curves randomly chosen. LoD was defined as the lowest RNA concentration where all replicates were detected. LoQ was defined as the lowest concentration where the relative standard deviation (RSD) was < 30%. The standard curve parameters for the RT-qPCR assay such as the y-intercept (*b*), the slope (*m*), the linearity (*R*^2^), and the amplification efficiency (*E*) were also calculated following the recommendations by the MIQE guidelines (Bustin et al. 2009).

### Models and data analysis procedure

#### Statistical smoothing methods

The use of nonparametric regression models for data smoothing is necessary to describe and understand the evolution of the pandemic. The fitting of these models is useful to identify outbreaks days from observing variables such as the viral load. The nonparametric approaches applied in this work were the Generalized Additive Models (GAM) (Wood 2017), the Locally Estimated Scatterplot Smoothing (LOESS) (Cleveland 1979), kernel regression (Wand and Jones 1994), local polynomial regression (Fan and Gijbels 1996 ), and the local bandwidth nonparametric regression (Herrmann and Maechler 2021).

GAM models are used given their flexibility to reproduce complex trajectories without involving experimental error fitting as a function of linear and smooth effects of the explanatory variables on the response (Vallejo et al. 2022). Thus, the expression *Y* = *β*_*0*_ + *β*_*1*_* X* + *s*(*T*) + *ε* shows that the response variable $Y$ can be expressed as a function of a linear effect of $X$ and a smooth effect on the predictor $T$, with ${\beta }_{0}$ and ${\beta }_{1}$ being the parameters defining the linear effect, $s\left(T\right)$ the smooth effect, and $\varepsilon$ the random error. In the present case, the smooth effect of time on viral load and the number of identified infected persons have been estimated, $E(\mathrm{viral load}|\mathrm{tim}e)=s\left(\mathrm{time}\right)$ and $E({N}^{\underset{\_}{^\circ }} \mathrm{of infected persons}|\mathrm{time})=s\left(\mathrm{time}\right)$ by the fitting of spline bases whose expression can be summarized by $s\left(t\right)=\sum_{k=1}^{L+\mathrm{degree}}{\beta }_{k}{\phi }_{k}\left(t\right)={\beta }^{^{\prime}}\phi$, defined by a weighted sum of *L* (number of knots in the time interval) + degree (degree of the splines) elements, in which ${\phi }_{k}\left(t\right)$ are the spline functions. In this work, thin plate regression splines (fewer parameters to optimize), cubic regression splines with shrinkage (that include a penalty on the second derivative), and Gaussian process smooths (high flexibility) are used (Wood 2017).

A popular fitting alternative was the use of the local LOESS regression method (Cleveland 1979) incorporating two regression or local smoothing models, i.e., kernel and linear local polynomial. Kernel regression is also applied. Assume that the regression function is$r$, and ${Y}_{i}$(with $i = 1, ..., n$) are the $n$ observations of the $Y$ response variable. Thus, $Y$ can be expressed as a function of the independent variable: ${Y}_{i}= r({t}_{i}) + {\varepsilon }_{i}$, with ${\varepsilon }_{i}$ the independent mean-zero error terms and ${t}_{i}$ the values of the $t$ design variable where the model is evaluated. In this case, the regression function can be estimated using kernel methods, such as Gasser and Müller method (Gasser et al. 1991),$\widehat{r}(t;h) = \frac{1}{h} \sum_{i=1}^{n}\left({g}_{i}\left(t;h\right)\cdot {Y}_{i}\right)$, where${g}_{i}\left(t;h\right)=\underset{{s}_{i}-1}{\overset{{s}_{i}}{\int }}W\left[(t-u)/h\right]du$, *s*_*i*_ = (t_i_ + t_i+1_)/2 with${s}_{0}=0$,${s}_{n}=1$, $W$ is the kernel function (Gaussian, Epanechnikov, etc.), and *h* is the smoothing parameter, also called bandwidth. The latter is the expression used in the *lokern* package (Herrmann and Maechler 2021; R Core Team 2022), which also includes a plug-in method to estimate locally the bandwidth (Herrmann 1997). The optimal bandwidths are obtained by minimizing an estimate of the asymptotically optimal mean squared error (Herrmann and Maechler 2021).

On the other hand, the local linear regression estimator is also applied (Francisco-Fernández et al. 2015). Consider ${\{({t}_{i},{Y}_{i})\}}_{i=1}^{n}$ the observed values of a curve of viral load or number of infected persons, ${Y}_{i}$, evaluated at different times, ${t}_{i}$, with $i=\mathrm{1,2},\dots ,n$, thus the he response variable can be expressed as a sum of the regression function $r({t}_{i})$ as follows, ${Y}_{i}=r({t}_{i})+{\varepsilon }_{i}$, with $1\le i\le n$ and ${\varepsilon }_{i}$ are the random errors. Of course, the aim is smoothing the data to reduce the noise of the experiment. This is done by fitting the data via local polynomial regression with a $p$-degree polynomial as shown by the expression ${\widehat{r}}_{h}(t)={{e}^{^{\prime}}}_{1}{({{X}^{^{\prime}}}_{t}{U}_{t}{X}_{t})}^{-1}{{X}^{^{\prime}}}_{t}{U}_{t}Y$, with ${e}_{1}={(\mathrm{1,0},\dots ,0)}^{^{\prime}}$, $Y={({Y}_{1},\dots ,{Y}_{n})}^{^{\prime}}$, $${X}_{t}=\left[\begin{array}{c}1\\ \vdots \\ 1\end{array}\begin{array}{c}\left({t}_{1}-t\right)\\ \vdots \\ \left({t}_{n}-t\right) \end{array}\begin{array}{c}\cdots \\ \\ \cdots \end{array}\begin{array}{c}{\left({t}_{1}-t\right)}^{p}\\ \vdots \\ {\left({t}_{n}-t\right)}^{p}\end{array}\right]$$ and ${U}_{t}=diag\{W(({t}_{1}-t)/h),\dots ,W(({t}_{n}-t)/h)\}$, while $W(\cdot )$ is a kernel function, $p$ the degree of the local polynomial ($p=1$ for the local linear), and $h$ the bandwidth. The bandwidth $h$ is crucial to obtain a proper fit. In the specific case of the viral load fitting by a linear local polynomial, the bandwidth is locally estimated by using cross-validation (Vieu 1991).

The three alternatives for smoothing have provided a way to properly estimate the trend of viral load of COVID-19 in wastewaters. For the application of these techniques, the following R packages (R Core Team 2022), *mgcv* (Wood 2017), *lokern* (Herrmann and Maechler 2021), *sm* (Bowman and Azzalini 2021), and *kernsmooth* (Wand 2021), among others, have been used.

#### Estimation of the real number of infected people from SARS-CoV-2 viral load in wastewater

Statistical models previously developed by our team and described in detail in Vallejo et al. (2022) were used to estimate the positive cases in the metropolitan area of A Coruña served by the WWTP using the proportion of cumulative positive cases in the same area from the viral load data detected in wastewater. The population reported for each sampler has been obtained from the INE (Spanish National Statistics Institute). More concretely, data by census sections was first obtained from the statistics of the Continuous Register Statistics on January 1, 2019 (INE 2019), the last data available, and then, the census sections’ data corresponding to the sewage subnetwork of the sampler was aggregated.

#### Estimation of the frequency of SARS-CoV-2 variants

To estimate the frequency of the SARS-CoV-2 VOCs, we have previously implemented a maximum likelihood model that is described in detail in López de Ullibarri et al. (2023).

#### Bioinformatic analysis

Single-nucleotide variants (SNVs) and indels were identified with iVar (Grubaugh et al. 2019). We aligned the paired-end reads to the reference sequence MN908947.3 from Wuhan using the BWA-mem tool (Li 2013) and sorted them using SAMtools (Danecek et al. 2021). Then, iVar *trim* was used to soft-clip the primer sequences and low-quality bases and remove reads shorter than 30 bp. Then, iVAR *consensus* was applied, with a minimum frequency threshold of 0.5. In addition, iVar functions *getmasked* and *removereads* were used to remove the reads corresponding to the indexes of the mismatched primers. SAMtools *mpileup* and iVar *variants* were used to identify single nucleotide changes and indels. A minimum frequency threshold of 0.0001 and a minimum depth of 1 were set for running iVar. The minimum base quality score threshold was left as default, 20. Then, iVar’s output was processed in R software (R Core Team 2022) to detect typical mutations of specific SARS-CoV-2 variants, which were obtained from the website http://outbreak.info.

#### Open access data sharing

A public website was developed to show the current epidemiological situation over time in A Coruña (Spain), available at https://edarbens.es/covid19/. The Leaflet library (Agafonkin 2020) was used to include maps showing the historical viral load (copies of viral RNA per L) in the different districts over time. These maps use viral load data included as JSON on the website. Daily estimations of the viral load are also publicly available on the website. COVIDBENS is included in the NORMAN SCORE “SARS-CoV-2 in sewage” (SC2S) database (NORMAN 2021), an international platform for rapid, open access data sharing about wastewater pathogen surveillance.

## Results

### RT-qPCR standard curve characteristics

LoD and LoQ values of the RT-qPCR assay were determined using 20 replicates of serial dilutions of EVAg standard ranging from 5 to 500 RNA copies/µL. Cq values and detection rates are shown in Table S1 (Online Resource 1), showing that 10 copies/µL is the LoD and 5 copies/µL is de LoQ. The slope of the standard curve for the N gene was − 3.1709, the y-intercept was 41.134, the R^2^ value was 0.9918, and the amplification efficiency was above 106.71% (Online Resource 2).

### Evolution of SARS-CoV-2 viral load over time

For the present study, viral load was analysed over 22 months in the metropolitan area of A Coruña (Spain). The COVIDBENS monitoring program collected a total of 863 samples of sewage from the Bens WWTP and from the municipalities of A Coruña, Arteixo, Culleredo, Oleiros, and Cambre. RT-qPCR results of viral load (copies of viral RNA per L) and Cq values obtained for the N gene are given in Online Resource 3.

**Fig. 2:**
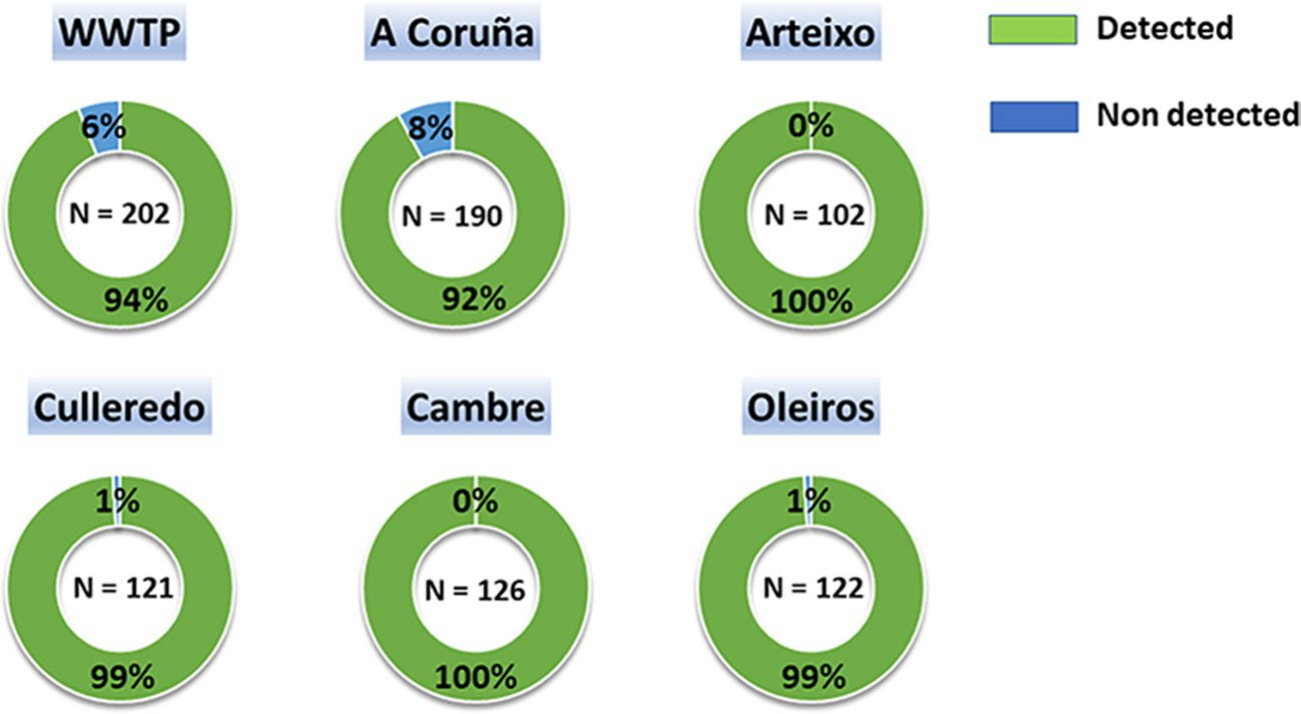
Detection rates of SARS-CoV-2 in wastewater samples of the different sampling points located in the metropolitan area of A Coruña (Spain). N represented the number of collected samples

**Fig. 3:**
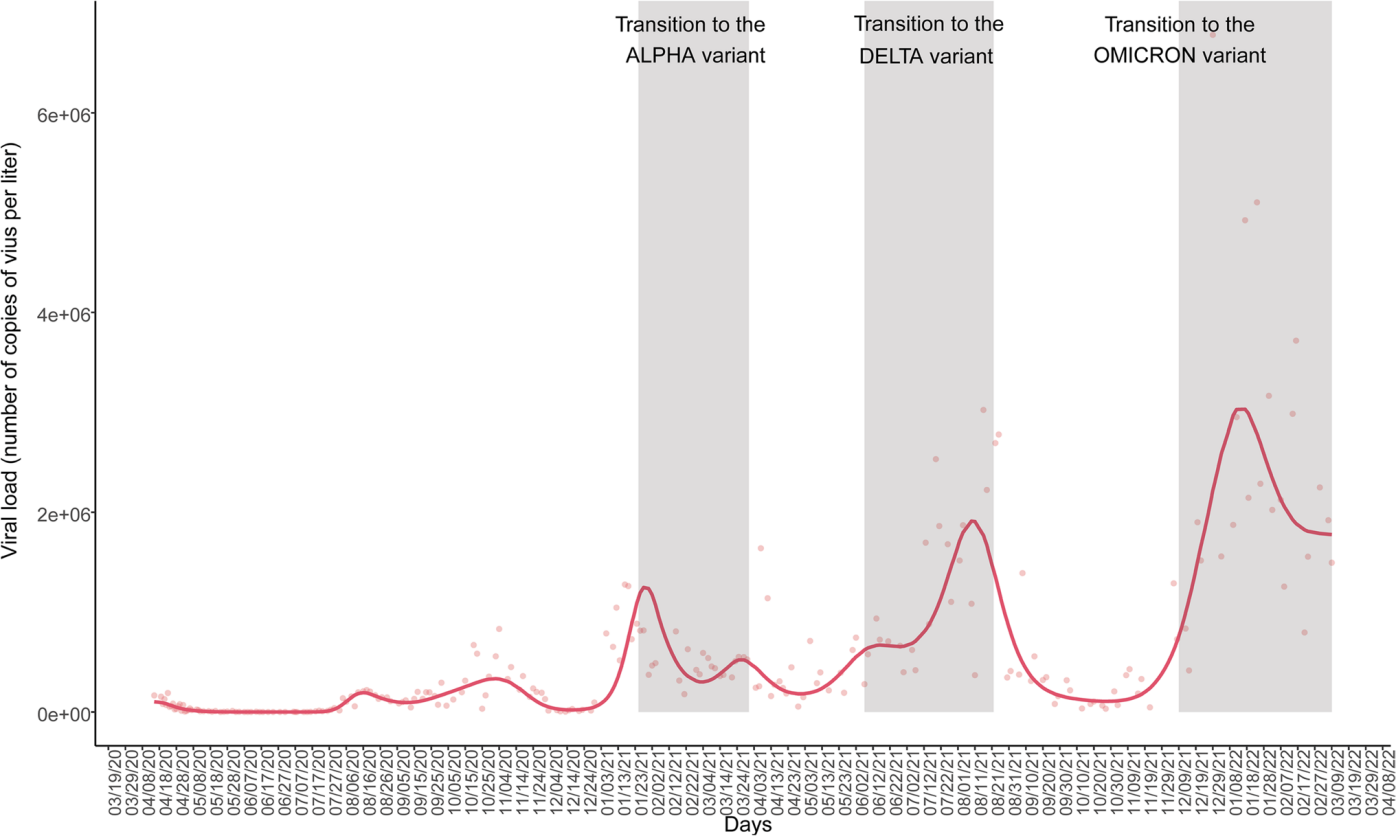
Evolution of the SARS-CoV-2 viral load measured at the Bens WWTP (A Coruña, Spain). The transition to each new variant is indicated with grey shadows

**Fig. 4:**
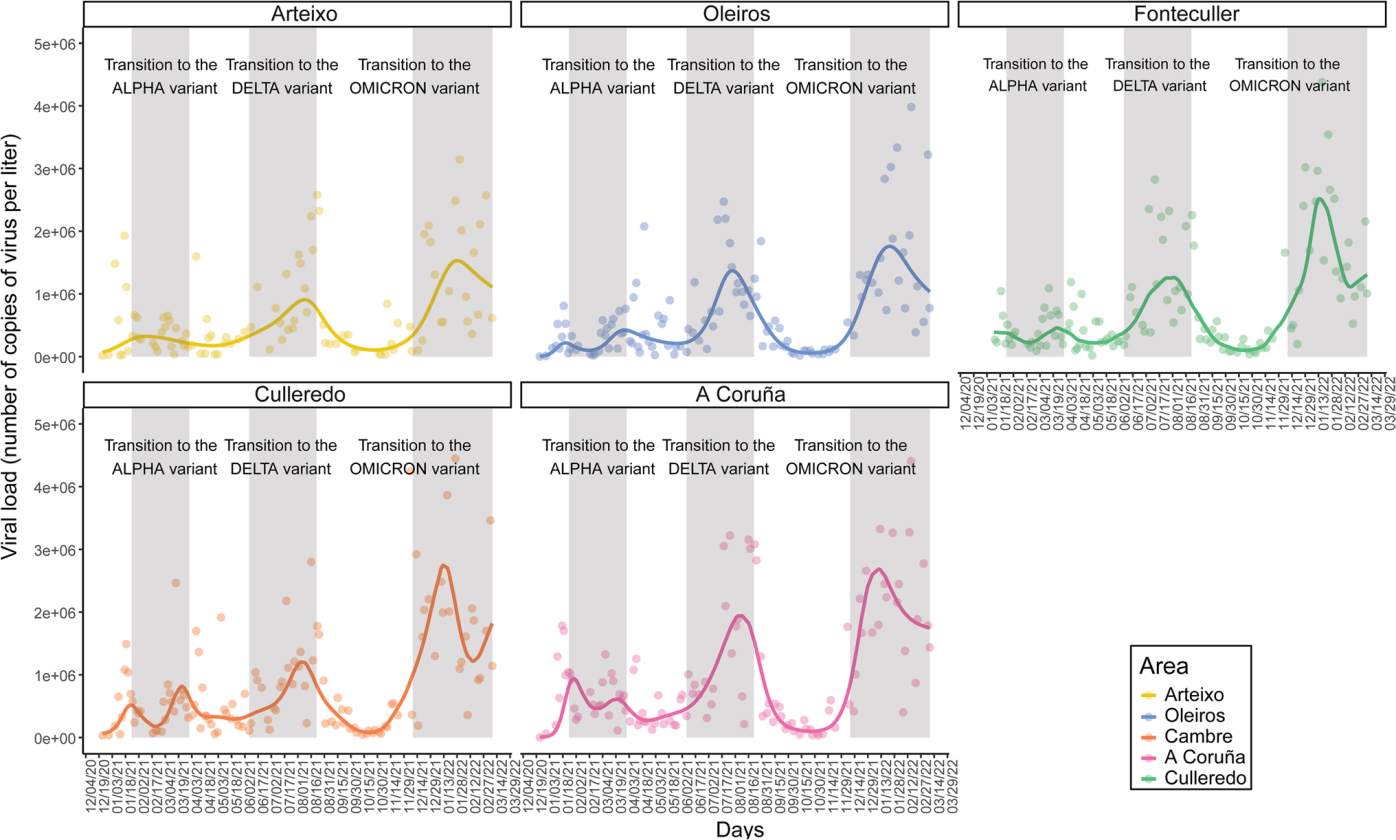
Evolution of the SARS-CoV-2 viral load in samples obtained from sewage from the municipalities of A Coruña, Arteixo, Cambre, Culleredo, and Oleiros from December 19, 2020 to March 17, 2022. The transition periods to new variants are indicated with grey shadows

SARS-CoV-2 RNA was detected in 96.5% of the wastewater samples. Detection rates of the viral RNA of the different sampling points are represented in Fig. 2. Viral load calculated from the N gene ranged from 1.30 × 10^3^ to 5.87 × 10^6^ copies/L (Table 1). The detection of SARS-CoV-2 in wastewater in the metropolitan area of A Coruña, including the five municipalities, collected in the WWTP of Bens (Fig. 3) showed six viral load waves in wastewater after the first COVID-19 outbreak reported in March 2020 (Vallejo et al. 2022). After the lockdown and the imposition of new restriction measures by the government the lowest viral load in wastewater was obtained, failing to amplify between June and July 2020. Nevertheless, in July 2020 the viral load increased again beginning the first wave, obtaining a maximum of 218.755 copies per L. The second wave covered the period from September to November 2020, where a maximum of 831.309 copies per L was obtained. After this second wave, viral load in wastewater increased again making way for the third wave that began with the emergence of the Alpha variant (B.1.1.7) in January 2021, reaching a maximum of 1.276,329 copies per L. Then, the viral load decreased again until the emergence of the fourth wave, where a peak between March and April 2021 was detected. Although the viral load values remained high, a fifth wave was observed from May to September 2021, where viral load reached around 3 million copies per L due to the appearance of the Delta variant (B.1.617.2). The last wave reported in this study is represented by the progressive increase in viral load from November 2021 to February 2022 due to the irruption of Omicron variant (B.1.1.529), obtaining a maximum of 5.102.970 copies per L. The evolution of the viral load measured separately in the different municipalities, which started some months later than in the whole area, followed a similar trend (Fig. 4), being consistent with that observed at the WWTP of Bens.

**Table 1:** Summary of SARS-CoV-2 monitoring results at the different sampling points in the metropolitan area of A Coruña (Spain) from June 2020 to March 2022

Area	Population in number of inhabitants	SARS-CoV-2 detection rate	SARS-CoV-2 viral load range (copies/L)
WWTP	369,098	94% (*N*^a^ = 200)	1.30 × 10^3^–5.10 × 10^6^
A Coruña	166,279	91% (*N* = 188)	1.68 × 10^3^–5.87 × 10^6^
Arteixo	30,942	100% (*N* = 102)	1.56 × 10^4^–5.85 × 10^6^
Culleredo	23,101	99% (*N* = 121)	2.00 × 10^4^–4.38 × 10^6^
Cambre	30,006	100% (*N* = 126)	1.18 × 10^4^–5.84 × 10^6^
Oleiros	33,663	99% (*N* = 122)	1.25 × 10^4^–3.98 × 10^6^

*WWTP*, wastewater treatment plant

^a^Refers to the total number of collected samples

### COVIDBENS as an early warning system

**Fig. 5:**
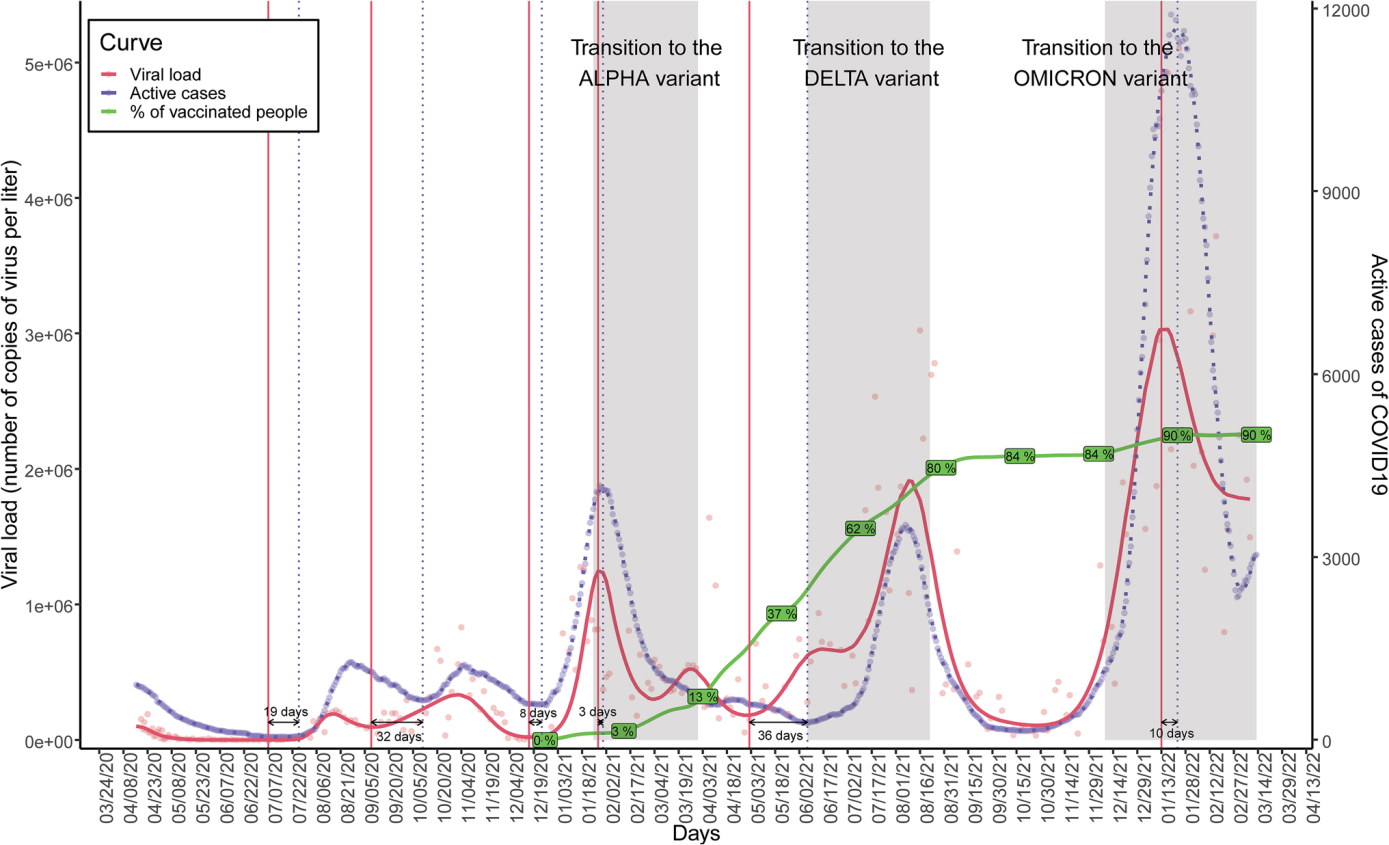
Representation of the early warning system as assessed by COVIDBENS. Smoothed viral load measured at the Bens WWTP (red line), official number of active cases (blue line), reported by the health authorities (XUGA, Spain), and percentage of vaccinated population (green line) in the metropolitan area of A Coruña (Spain). Vertical dotted lines highlight increases of viral load (red) and clinical cases (blue). The transition periods to new emerging variants are indicated with grey shadow

SARS-CoV-2 viral load measured at the Bens WWTP was plotted and compared with the number of active clinical cases reported by the health authorities in A Coruña (Fig. 5). The comparison revealed that an increase in viral load in wastewater clearly preceded a subsequent increase in clinical cases in each of the successive pandemic waves. Official COVID-19 cases reported by the health authorities are given in Online Resource 4. Remarkably, increases in viral load corresponding to the different epidemic waves were detected in wastewater before the clinical outbreaks were reported (Fig. 5) with 8–36 days of anticipation (Table 2).

**Table 2:** SARS-CoV-2 detection results and prediction of COVID-19 clinical cases (lead time) in this study (A Coruña, Spain) from June 2020 to March 2022, and in other studies

Reference	Location	Sampling data	Maximum concentration	Lead time
This study	A Coruña (Spain)	1 WWTP and 6 sewers; composite samples; 21 months	10^6^ copies per L	36 days
Yaniv et al. 2021	Ashkelon (Israel)	1 WWTP and 9 sewers; composite samples; 4 days	10^7^ copies per L	ND
Kumar et al. 2021	Ahmedabad (India)	1 WWTP and 8 sewage pumping stations; grab samples; 13 weeks	10^4^ copies per L	1–2 weeks
Chavarria-Miro et al. 2021	Barcelona (Spain)	2 WTTP; composite samples; 3 months	10^5^ copies per L	41 days
Reynolds et al. 2022	Dublin (Ireland)	1 WWTP; composite samples; 14 months	1.5e + 14 genome copies/day	0 days

*ND*, not determined

*WWTP*, wastewater treatment plant

**Fig. 6:**
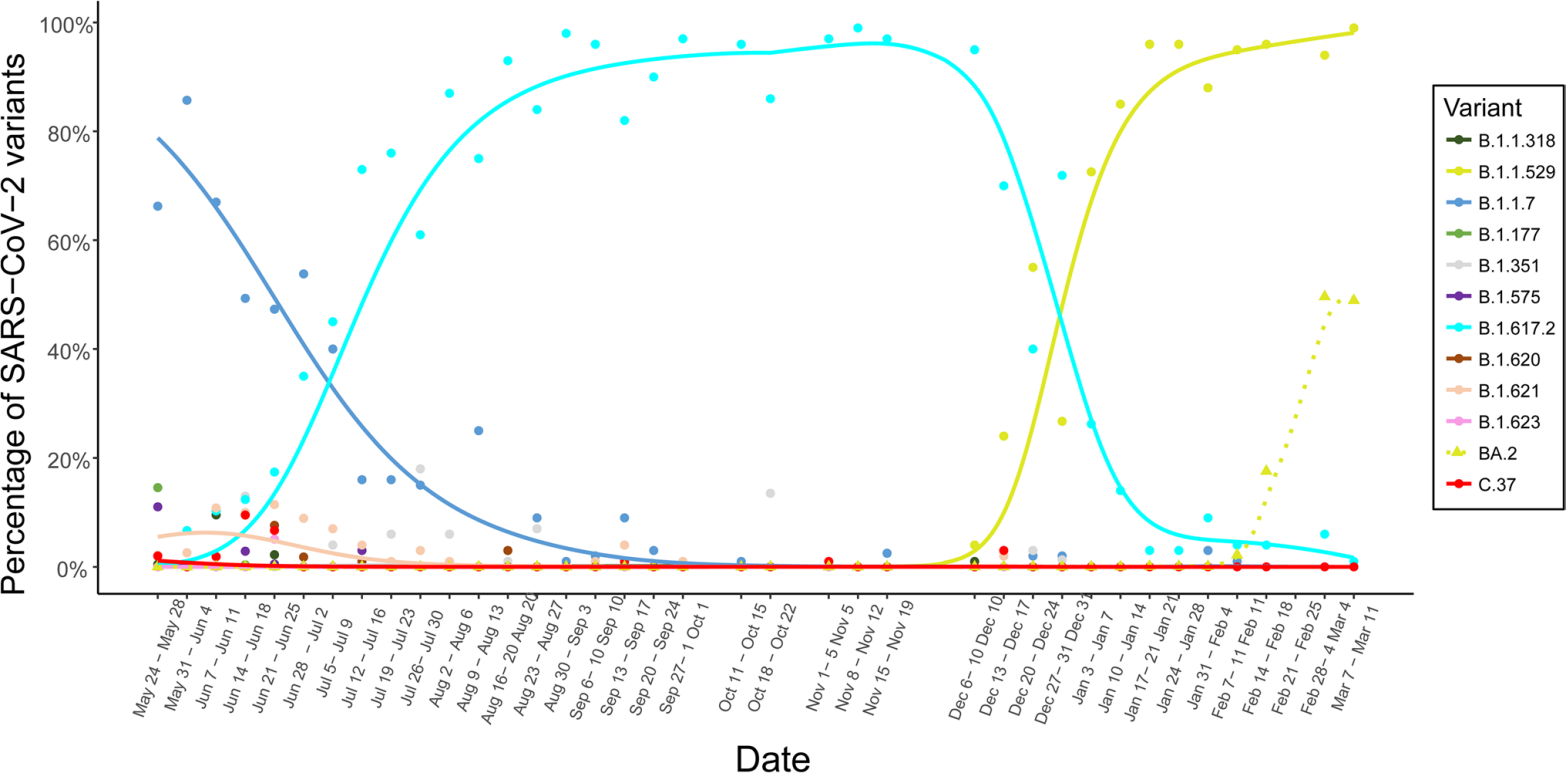
Evolution of the SARS-CoV-2 variant frequency over time based on mutations detected in wastewater samples from the metropolitan area of A Coruña (Spain) from May 2021 to March 2022 using statistical models previously described. Arrows indicate the first clinical case reported by the health system infected with each variant (data recovered by the Galician Health Service, SERGAS)

The monitorization of SARS-CoV-2 mutations in wastewater was carried out from November 2020 to March 2022. Moreover, the evolution of variants’ frequency in the local population over time was determined from May 2021 to March 2022 (Fig. 6) using the statistical method previously described by our team (López de Ullibarri et al. 2023). We detected the Alpha variant in the wastewater samples on December 16, 2020, 42 days before it was detected in the clinical samples (January 27, 2021) (Fig. 6), being the longest lead time achieved here (Table 3). Thus, Alpha was the predominant variant during the third viral load wave. Similarly, we detected the Delta variant in wastewater on May 18, 2021, 30 days before the appearance of clinical cases (June 17, 2021). Delta became predominant during the fifth viral load wave. After that, the Delta abundance in wastewater decreased progressively until it was replaced by the Omicron variant in January 2022. Finally, we could also anticipate the emergence of the Omicron lineage BA.2, detected in wastewater on January 18, 2022, 27 days before its identification in clinical samples on February 14, 2022.

**Table 3:** SARS-CoV-2 variants predicting results in this work (A Coruña, Spain) from November 2020 to March 2022, and in other studies

Reference	Location	Sampling data	Variant detected	Variant prediction
This study	A Coruña (Spain)	1 WWTP and 6 sewers; composite samples; 21 months	Alpha, Delta, and Omicron	42 days
Joshi et al. 2022	Ahmedabad (India)	2 WTTP and 1 river; grab samples; 3 months	Delta	1 month
Vo et al. 2022	Las Vegas (EEUU)	7 WTTP; grab and composite samples; 1 year	Alpha and Epsilon	1 month
Jahn et al. 2022	Zurich, Lausanne, and alpine ski resort (Swiss)	3 WWTP; grab and composite samples; 8 months	Alpha	13 days
Karthikeyan et al. 2022	San Diego (California)	1 WWTP and campus sewers; composite samples; 1 year	Alpha and Delta	14 days
Kirby et al. 2022	California, Colorado, New York, Texas (EEUU)	Multiple sewersheds; 1 month	Omicron	1 week

*WWTP*, wastewater treatment plant

### Evolution of the estimated number of infected people over time

**Fig. 7:**
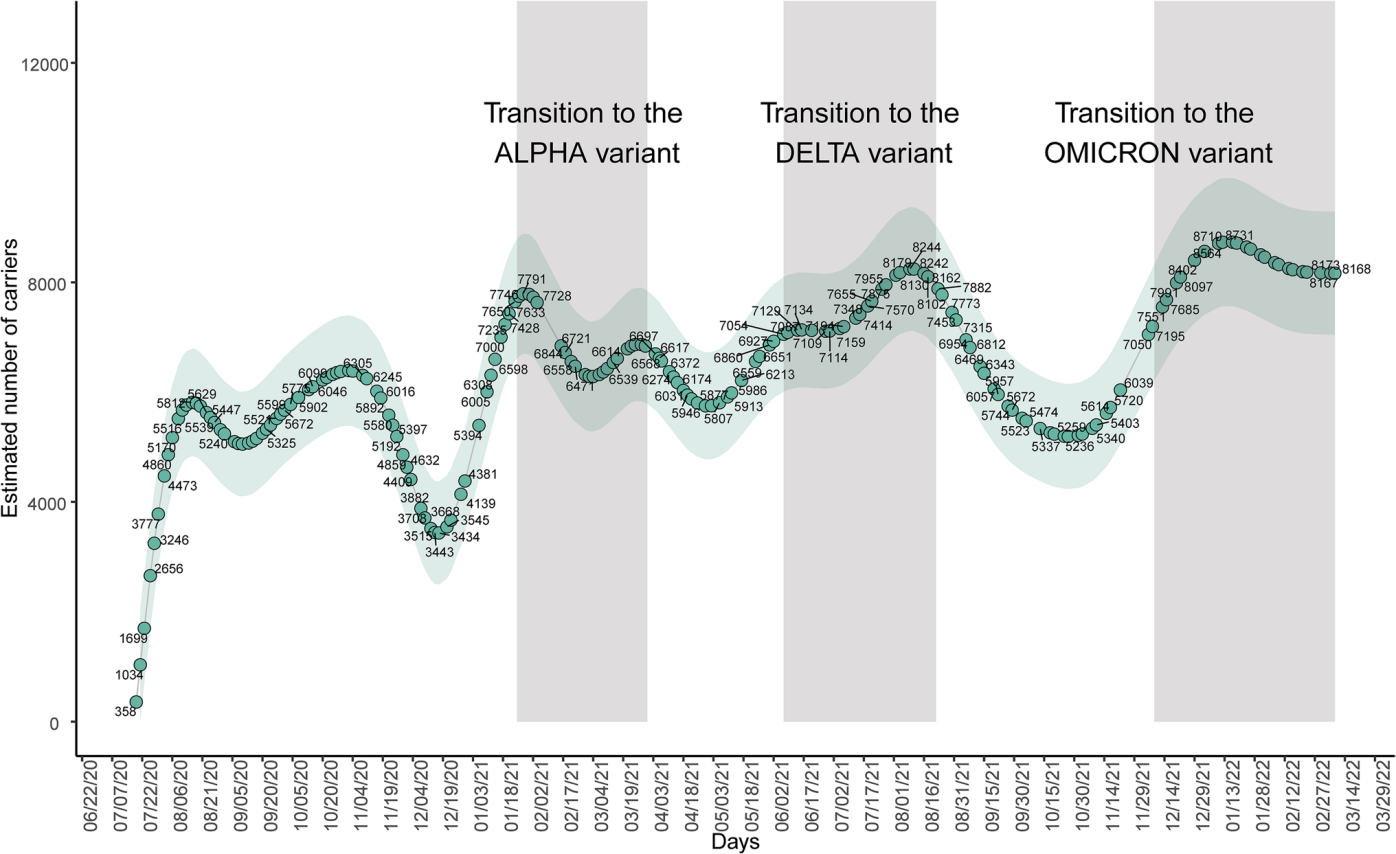
Evolution of the number of people infected by SARS-CoV-2 in the metropolitan area of A Coruña (Spain) during the pandemic from July 2020 to March 2022. Data have been estimated using COVIDBENS statistical models (Vallejo et al*.* 2022)

Statistical models previously developed by our team (Vallejo et al. 2022) were leveraged to estimate the number of people infected by SARS-CoV-2 over time in the successive epidemic waves (Online Resource 5), including symptomatic and asymptomatic people. The SARS-CoV-2 viral load in wastewater (Fig. 3) and the estimated number of infected people followed similar trends (Fig. 7) and peaks coincided with the emergence of SARS-CoV-2 variants.

## Discussion

Although many efforts have been made by researchers all over the world to detect asymptomatic people infected with SARS-CoV-2, using nasopharyngeal PCR or antigen and antibodies tests, to date, we are still not able to identify the entire population infected and, therefore the real magnitude of the epidemic is difficult to understand. Wastewater monitoring stands as a powerful tool to monitor the COVID-19 epidemic in the entire infected population, including symptomatic and asymptomatic people.

SARS-CoV-2 was absent in wastewater during the period June-July 2020, which can be explained by the efficacy of the hard restriction measures implemented from mid-March to the end of June. But, unfortunately, at the beginning of July the first new epidemic outbreak after restrictions occurred, followed by a long succession of epidemic waves. During the period of the present study, the maximum viral load detected in wastewater samples ranged from 10^5^ to 10^6^ RNA copies per L, similar to that reported in other studies (Gerrity et al. 2021; Padilla-Reyes et al. 2022; Pillay et al. 2021; Saththasivam et al. 2021). However, Yaniv et al. (2021) showed higher viral load values, around 10^7^ RNA copies per L, which could be related to the length of their study (4 days) and to the type of sample collecting (8 h composite samples). On the contrary, Kumar et al. (2021) reported a low viral load range, 10^4^ RNA copies per L, probably due to the use of grab samples, which, as reported by Rafiee et al. (2021), makes difficult to follow the evolution of viral load or to perform statistical approaches. Wastewater epidemiology is influenced by many factors such as the SARS-CoV-2 shedding pattern of each variant, the population size of each location, the physico-chemical characteristics of the wastewater, and others such as the commercial activity, or the international trade within and between countries, which induces variability in viral load data. Accordingly, the shedding of SARS-CoV-2 RNA in the human body depends on the stage of the disease in which the patient is. Wu et al. (2022) demonstrated an early SARS-CoV-2 shedding peak before symptom onset with a subsequent progressive reduction in RNA load in the last stages of the disease. The age also affects viral shedding, in such a way that a higher viral load is detected in older people (Vellas et al. 2020). Moreover, it can be established that the recovery of COVID-19 patients implies less shedding. Also, the total population covered by the sampling area is a crucial factor in WBE since the larger the population, the greater the flow of wastewater, thus diluting the viral RNA that reaches the WWTP (Wilder et al. 2021). It also should be taken into account that tourism and travel, which significantly change during summer, vary the population size. In addition, wastewater properties such as pH, temperature, humidity, ammonia or solid composition, or chlorination are directly associated with SARS-CoV-2 viral load measured in sewage samples (Ahmed et al. 2020; Amoah et al. 2022; Zhang et al. 2020). SARS-CoV-2 detection differs between the solid and the liquid phase of the wastewater influent, reporting a higher viral load in the liquid fraction (Weidhaas et al. 2021). In our previous work (Vallejo et al. 2022), we suggested that a high flow rate due to the rainfall and long transit times through the sanitary network until the WWTP, reduced SARS-CoV-2 concentration in wastewater, because the viral RNA was exponentially degraded during transportation. Therefore, the population living near the WWTP is probably the main contributor to the viral load detected in wastewater. The dilution effect of rainfall reduces SARS-CoV-2 RNA levels in wastewater. Saingam et al. (2023) used chemical indicators (phosphate and ammonia) to calibrate this dilution effect improving the correlation between viral load in wastewater and clinical data over time. Lastly, one of the factors that influence the amount of SARS-CoV-2 in wastewater and the detection of new mutations is international trade. This factor is a good indicator of the spread of the COVID-19 disease, since the most active areas in trade have greater population mobility and more group activities, increasing interpersonal contacts. Thus, social interactions are the main source of the spread of SARS-CoV-2 in society and it is correlated with confirmed clinical cases (Bontempi and Coccia 2021). In addition, international trade promotes the contact with foreign populations and consequently increases the risk of SARS-CoV-2 transmission between communities (Bontempi et al. 2021). These indicators help in the design of new effective policy responses to reduce the impact of future pandemics on society.

COVIDBENS was able to anticipate up to 36 days’ increases in clinical cases, or epidemic waves. Similar WBE studies resulted in 7–24 days of anticipation (Claro et al. 2021; Kumar et al. 2021; Robotto et al. 2022; Sangsanont et al. 2022). Remarkably, in Barcelona, Chavarria-Miro et al. (2021) reported an outbreak 41 days before the first clinical sample was detected. They performed different RT-qPCR assays targeting 5 SARS-CoV-2 genes instead of only one, increasing the robustness of the data. In other studies, despite using 24-h composite samples over a long surveillance period, no lead time was observed, as in the study reported by Reynolds et al. (2022). The ability to anticipate to early outbreaks depends on the sampling strategy used, the frequency of sampling and on the smoothing statistical methods used, among other factors. It has to be taken into account that, in general, limited data or punctual analyses do not allow establishing a reliable relationship between viral load and clinical cases using statistical models. COVIDBENS recruited samples at the Bens WWTP including all the municipalities of the metropolitan area of A Coruña, but also monitored the wastewater from specific locations, which was essential to detect local outbreaks. Regarding the sampling frequency, we collected samples twice or three times per week, during the period of the present study, depending on the evolution of the epidemic. After a preliminary study, we chose 24-h composite sampling as the best scheme for monitoring the epidemic in the metropolitan area of A Coruña.

A large proportion of people infected by SARS-CoV-2 have no symptoms, so they are not usually detected and thus reported by the health system. The statistical model used in this study (Vallejo et al. 2022) allowed us to estimate the proportion of people infected in the metropolitan area of A Coruña. However, this model was developed when lineage B.1.177 was prevalent in Spain. After that, several new VOCs have emerged with potentially larger viral shedding and consequently higher load in the wastewater. This fact requires an adaptation of the statistical models to the new situation of higher viral load excreted per person as new variants appear. Several studies confirmed the longer duration of Alpha variant in the respiratory tract of infected people resulting in higher RNA load compared to previous lineages (Calistri et al. 2021; Lyngse et al. 2021). Similarly, both Delta and Omicron variants seem to result in higher RNA load per patient than earlier variants (Riediker et al. 2022). Other authors suggested that the Omicron was more transmissible and contagious than Delta, but nevertheless they conclude that this variant does not generate a higher viral load per person than Delta (Chen et al. 2022; Migueres et al. 2022; Sentis et al. 2022). This explains the higher viral load values observed in wastewater in January 2022 due to the rapid spread of Omicron in the population. Therefore, epidemiological surveillance models for SARS-CoV-2 in wastewater must consider these differences in the levels of excretion of VOCs. Results of the estimated number of people infected by SARS-CoV-2 until January 2021 are 90% reliable as described by Vallejo et al. (2022), but from then on, a specific model adjustment would be necessary for each new variant emerged over time.

A relevant problem in the context of the COVID-19 pandemic was the monitorization of the progression of SARS-COV-2 variants. The most used classification tools, such as pangolin, were designed for clinical samples in which only one lineage predominates, but in case of wastewater samples, several variants coexist at the same time, which makes the bioinformatics analysis harder. SARS-CoV-2 genome sequencing from wastewater allows the detection of emerging variants far before they appear in clinical samples. We reported the Alpha, Delta, and Omicron variants up to 42 days earlier than the clinical cases detected by the regional health system. This anticipation demonstrates the great potential of genomic surveillance in wastewater samples. Ideally, for best WBE, variant detection and viral load monitoring in wastewater over time should be investigated in a complementary way (Galani et al. 2022; Johnson et al. 2022; Masachessi et al. 2022; Rubio-Acero et al. 2021). Few studies reported similar lead times (Joshi et al. 2022; Vo et al. 2022) while other achieved shorter lead times, around 7 days (Kirby et al. 2022). The genomic surveillance method used by Kirby et al. (2022) does not allow knowing if all the variant-associated mutations are present in a single genome, making difficult to confirm the presence of a specific variant in the wastewater sample. However, the statistical methods previously developed by our team (López de Ullibarri et al. 2023), used in the present study, allowed the determination of the percentage of each variant in the population over time from mutations data obtained from pool samples such as wastewater. More and more studies are implementing bioinformatic tools and statistical approaches for the early detection of low-frequency variants and the quantitative monitoring (Jahn et al. 2022). Karthikeyan et al. (2022) developed software for SARS-CoV-2 variants differentiation from wastewater samples. In addition, it was shown that the combined use of arctic v4 primers and the Maxwell® RSC Enviro Wastewater TNA Kit (TNA) increases the sensitivity and genome coverage of the virus sequencing (Girón-Guzmán et al. 2023).

The irruption of the Delta variant in the summer of 2021 had a much greater impact on viral load in wastewater than the Alpha variant had, probably due to its mutations in the spike protein which highly impact in its transmissibility and immune evasion (Tian et al. 2021). However, the number of COVID-19 reported cases during the Delta wave was lower, reflecting the vaccination effectiveness. The transition to the Omicron variant corresponds to the last viral load wave reported in this study. Despite the high vaccination rate in A Coruña (above 90%), the higher transmission rate of Omicron compared to Delta (Allen et al. 2022; Kumar et al. 2022) and its immune escape ability (Zhang et al. 2021) probably explain a larger viral load in the wastewater, in contrast to the low rate of hospitalized people (data not shown), which indicates the benefit of vaccines. Omicron continues to accumulate mutations, emerging several lineages which have rapidly spread globally during 2022 (Parums 2022). The BA.2 sublineage became the dominant variant at the end of this monitoring program due to its higher transmissibility and its capacity to reduce the effect of vaccination (Lyngse et al. 2022). Currently, the XBB.1.5 Omicron, detected for the first time in the USA, became predominant in Spain. The great impact of the VOCs in the population and their capability of reducing the protective effect of the vaccines increased the transmissibility of the infection, reporting a higher proportion of COVID-19 cases. However, hospitalization and mortality in COVID-19 cases were significantly reduced as the percentage of vaccinated people increased. This demonstrates the effectiveness of mass vaccination campaigns to contain the COVID-19 pandemic.

SARS-CoV-2 surveillance in wastewater in A Coruña helped in decision-making at both the political and hospital levels. Both the regional and local government used COVIDBENS data to implement the corresponding restriction measures, and hospitals were able to anticipate and prepare for an increase of cases. In addition, important companies have followed our data to plan their production according to the COVID-19 pandemic, suggesting that industry, among other sectors, can benefit from the implementation of these epidemiological models. Nevertheless, in addition to the implementation of an effective WBE system, it is essential to have effective and preventive strategies against future pandemic threats, not only focused on the health level but also on the social, environmental, and institutional levels. During the COVID-19 pandemic, it has been shown that measures such as the use of masks or lockdown considerably stopped the transmission of SARS-CoV-2 in the population. Other necessary preventive measures are the control of the transport and trade of pathogenic microorganisms, the reduction of air pollution levels in big cities, and transparent, responsive, and effective governance (Coccia 2021). Strategies such as contact tracing and clinical testing to diagnose SARS-CoV-2 infections in the population would reduce the transmission of the disease, minimizing the number of infected people and avoiding the greatest possible number of deaths, especially in the initial stages of a pandemic situation when effective drugs or vaccines are not yet available (Benati and Coccia 2022). In addition, the implementation of a rapid vaccination plan at the beginning of the pandemic, the negative impact of the pathogen on society would be reduced and future outbreaks and similar pandemics would be prevented (Coccia 2022a). For this reason, countries have to increase the R&D investment for the development of new effective vaccines and drugs. Coccia (2022b) developed the *r* (*resilience*) and *p* (*preparedness*) indexes, which measure the ability of countries to face the COVID-19 crisis based on their level of support for vaccine development and their mortality reduction policies. It is an effective method that could be implemented in prevention strategies for future similar pandemics. Even so, new improvements and scientific advances are still needed on WBE, not only focused on SARS-CoV-2 but also on other pathogenic microorganisms with pandemic potential (Núñez-Delgado et al. 2021).

Even though COVIDBENS monitoring program ended in March 2022, SARS-CoV-2 wastewater, we strongly believe that wastewater surveillance should continue for monitoring the COVID-19 pandemic. WBE is an effective tool with various applications that support public health actions (Prado et al. 2023): (i) provides information about the transmission of SARS-CoV-2 and other potentially dangerous pathogens; (ii) detects early outbreaks; (iii) allows monitoring the effectiveness of the measures imposed by governments and local authorities to contain the viral transmission; (iv) alerts about the emergence of new pathogens, such as Crimean Congo Haemorrhagic Fever virus or Monkeypox virus, recently considered in Europe; (v) serves to monitor antimicrobials resistance in the community; and (vi) helps political authorities to be prepared for future pandemics. For these reasons, the European Commission recommended the inclusion of WBE in the national detection strategies (EC 2021b).

## Limitations of the present study

Our study has some limitations that must be taken into account. First, we did not consider the dilution rate in wastewater. Flow normalization is commonly used when reliable flow data are available, but in the absence of these, alternative methods such as electrical conductivity and crAssphage can be very effective (Langeveld et al. 2023). Secondly, we assumed no variation in the shedding pattern along the surveillance period, but the different VOC excretion levels in feces affect both the early warning condition of the WBE for COVID-19 and the estimated infected people results. This could be related with a decrease in the ability to anticipate the emergence of Omicron with respect to Delta. As we described before, our statistical model to estimate the people infected with SARS-CoV-2 was only designed for the original variant (B.1.177), so it must be adjusted for new each new emerging variant over time. Thirdly, WBE has a low sensitivity and recovery of the SARS-CoV-2 detection in sewage due to the complexity of the wastewater samples, so new methods are needed to improve the sensitivity and virus recovery such as the EM-VIP-Mag-RT-qPCR method (Kumblathan et al. 2023). In addition, due to the distribution of the sewerage network, the specific location of the sewers installed in the five municipalities of the metropolitan area of A Coruña do not cover the entire population, which may affect the WBE system efficiency, given that we lost a small part of the infected population. A deeper redesign would be necessary to resolve this issue. Lastly, the use of automatic samplers is highly expensive, so it is not valid for all types of regions. Studies have demonstrated the efficacy of passive sampling in WBE in small areas using Moore’s swabs, which are more sensitive, precise, and cost-effective, so they could be implemented in low-resource areas where clinical testing is scarce (Cha et al. 2023).

## Conclusions

This work demonstrates that SARS-CoV-2 viral load monitoring in wastewater is a very efficient way to report on COVID-19 evolution in the community, anticipating new outbreaks long before the health system, and therefore allowing the public authorities to take reliable decisions, saving precious time and public resources. SARS-CoV-2 monitoring in wastewater detects increases in viral load before clinical cases appear being able to predict epidemic waves in the population. Viral load data combined with statistical models allows estimating the total number of infected people (symptomatic and asymptomatic), improving significantly the WBE strategy. In addition, sequencing of SARS-CoV-2 in wastewater allows a real-time epidemiological surveillance of SARS-CoV-2 mutations and combined with statistical models, can anticipate the appearance of emerging VOCs before clinical testing. COVIDBENS has served as an effective early warning system for anticipating new outbreaks and variants of concern in the metropolitan area of A Coruña and, it also provided a public health service open to all citizens with an important social impact throughout the entire pandemic. Nations should implement the WBE system in their prevention strategies for future pandemic crises, which also include social, environmental, and health policies, to reduce the socioeconomic impact of the virus or other potentially dangerous pathogens.

## Supplementary Information

Below is the link to the electronic supplementary material.

Supplementary file1 (DOCX 16 KB)

Supplementary file2 (DOCX 29 KB)

Supplementary file3 (XLSX 225 KB)

Supplementary file4 (XLSX 44 KB)

Supplementary file5 (XLSX 21 KB)

## Data Availability

All data used for this study are provided in this manuscript as supplementary files; ESM_1.docx, ESM_2. docx, ESM_3.xlsx, ESM_4.xlsx, and ESM_5.xlsx.

## References

[CR1] Acosta N, Bautista MA, Waddell BJ, McCalder J, Beaudet AB, Man L, Pradhan P, Sedaghat N, Papparis C, Bacanu A, Hollman J, Krusina A, Southern DA, Williamson T, Li C, Bhatnagar S, Murphy S, Chen J, Kuzma D, Clark R, Meddings J, Hu J, Cabaj JL, Conly JM, Dai X, Lu X, Chekouo T, Ruecker NJ, Achari G, Ryan MC, Frankowski K, Hubert CRJ, Parkins MD (2022). Longitudinal SARS-CoV-2 RNA wastewater monitoring across a range of scales correlates with total and regional COVID-19 burden in a well-defined urban population. Water Res.

[CR2] Agafonkin V (2020) Leaflet—An open-source JavaScript library for mobile-friendly interactive maps. Leaflet. https://leafletjs.com/reference1.6.0.html. Accessed 17 June 2021

[CR3] Ahmed W, Bertsch PM, Bibby K, Haramoto E, Hewitt J, Huygens F, Gyawali P, Korajkic A, Riddell S, Sherchan SP, Simpson SL, Sirikanchana K, Symonds EM, Verhagen R, Vasan SS, Kitajima M, Bivins (2020). Decay of SARS-CoV-2 and surrogate murine hepatitis virus RNA in untreated wastewater to inform application in wastewater-based epidemiology. Environ Res.

[CR4] Ahmed W, Simpson SL, Bertsch PM, Bibby K, Bivins A, Blackall LL, Bofill-Mas S, Bosch A, Brandão J, Choi PM, Ciesielski M, Donner E, D’Souza N, Farnleitner AH, Gerrity D, Gonzalez R, Griffith JF, Gyawali P, Haas CN, Hamilton KA, Hapuarachchi HC, Harwood VJ, Haque R, Jackson G, Khan SJ, Khan W, Kitajima M, Korajkic A, La Rosa G, Layton BA, Lipp E, McLellan SL, McMinn B, Medema G, Metcalfe S, Meijer WG, Mueller JF, Murphy H, Naughton CC, Noble RT, Payyappat S, Petterson S, Pitkänen T, Rajal VB, Reyneke B, Roman FA, Rose JB, Rusiñol M, Sadowsky MJ, Sala-Comorera L, Setoh YX, Sherchan SP, Sirikanchana K, Smith W, Steele JA, Sabburg R, Symonds EM, Thai P, Thomas KV, Tynan J, Toze S, Thompson J, Whiteley AS, Wong JCC, Sano D, Wuertz S, Xagoraraki I, Zhang Q, Zimmer-Faust AG, Shanks OC (2022). Minimizing errors in RT-PCR detection and quantification of SARS-CoV-2 RNA for wastewater surveillance. Sci Total Environ.

[CR5] Albert S, Ruíz A, Pemán J, Salavert M, Domingo-Calap P (2021). Lack of evidence for infectious SARS-CoV-2 in feces and sewage. Eur J Clin Microbiol Infect Dis.

[CR6] Allen H, Tessier E, Turner C, Anderson C, Blomquist P, Simons D, Løchen A, Jarvis CI, Groves N, Capelastegui F, Flannagan J, Zaidi A, Chen C, Rawlinson C, Hughes GJ, Chudasama D, Nash S, Thelwall S, Lopez-Bernal J, Dabrera G, Charlett A, Kall M, Lamagni T (2022) Comparative transmission of SARS-CoV-2 Omicron (B.1.1.529) and Delta (B.1.617.2) variants and the impact of vaccination: national cohort study, England. medRxiv 2022.02.15.22271001. 10.1101/2022.02.15.22271001

[CR7] Amin N, Haque R, Rahman MdZ, Rahman MZ, Mahmud ZH, Hasan R, Islam MT, Sarker P, Sarker S, Adnan SD, Akter N, Johnston D, Rahman M, Liu P, Wang Y, Shirin T, Rahman M, Bhattacharya P (2023). Dependency of sanitation infrastructure on the discharge of faecal coliform and SARS-CoV-2 viral RNA in wastewater from COVID and non-COVID hospitals in Dhaka, Bangladesh. Sci Total Environ.

[CR8] Amoah ID, Abunama T, Awolusi OO, Pillay L, Pillay K, Kumari S, Bux F (2022). Effect of selected wastewater characteristics on estimation of SARS-CoV-2 viral load in wastewater. Environ Res.

[CR9] Artic-network (2020) arctic-ncov2019. Github. https://github.com/artic-network/artic-ncov2019/tree/master/primer_schemes/nCoV-2019/V3. Accessed 3 November 2020

[CR10] Barua VB, Juel MAI, Blackwood AD, Clerkin T, Ciesielski M, Sorinolu AJ, Holcomb DA, Young I, Kimble G, Sypolt S, Engel LS, Noble RT, Munir M (2022). Tracking the temporal variation of COVID-19 surges through wastewater-based epidemiology during the peak of the pandemic: a six-month long study in Charlotte, North Carolina. Sci Total Environ.

[CR11] Benati I, Coccia M (2022). Effective contact tracing system minimizes COVID-19 related infections and deaths: policy lessons to reduce the impact of future pandemic diseases. J Public Adm Govern.

[CR12] Berchenko Y, Manor Y, Freedman LS, Kaliner E, Grotto I, Mendelson E, Huppert A (2017). Estimation of polio infection prevalence from environmental surveillance data. Sci Transl Med.

[CR13] Blomqvist S, El Bassioni L, El Maamoon Nasr EM, Paananen A, Kaijalainen S, Asghar H, de Gourville E, Roivainen M (2012). Detection of imported wild polioviruses and of vaccine-derived polioviruses by environmental surveillance in Egypt. Appl Environ Microbiol.

[CR14] Bontempi E, Coccia M (2021). International trade as critical parameter of COVID-19 spread that outclasses demographic, economic, environmental, and pollution factors. Environ Res.

[CR15] Bontempi E, Coccia M, Vergalli S, Zanoletti A (2021). Can commercial trade represent the main indicator of the COVID-19 diffusion due to human-to-human interactions? A comparative analysis between Italy, France, and Spain. Environ Res.

[CR16] Bowman AW, Azzalini A (2021) R package ‘sm’: nonparametric smoothing methods (version 2.2–5.7). University of Glasgow, Statistics. http://www.stats.gla.ac.uk/~adrian/sm. Accessed 20 June 2020

[CR17] Brouwer AF, Eisenberg JNS, Pomeroy CD, Shulman LM, Hindiyeh M, Manor Y, Grotto I, Koopman JS, Eisenberg MC (2018). Epidemiology of the silent polio outbreak in Rahat, Israel, based on modeling of environmental surveillance data. Proc Natl Acad Sci U S A.

[CR18] Bustin SA, Benes V, Garson JA, Hellemans J, Huggett J, Kubista M, Mueller R, Nolan T, Pfaffl MW, Shipley GL, Vandesompele J, Wittwer CT (2009). The MIQE guidelines: minimum information for publication of quantitative real-time PCR experiments. Clin Chem.

[CR19] Calistri P, Amato L, Puglia I, Cito F, Di Giuseppe A, Danzetta ML, Morelli D, Di Domenico M, Caporale M, Scialabba S, Portanti O, Curini V, Perletta F, Cammà C, Ancora M, Savini G, Migliorati G, D’Alterio N, Lorusso A (2021). Infection sustained by lineage B.1.1.7 of SARS-CoV-2 is characterised by longer persistence and higher viral RNA loads in nasopharyngeal swabs. Int J Infect Dis.

[CR20] Cha G, Graham KE, Zhu KJ, Rao G, Lindner BG, Kocaman K, Woo S, D’amico I, Bingham LR, Fischer JM, Flores CI, Spencer JW, Yathiraj P, Chung H, Biliya S, Djeddar N, Burton LJ, Mascuch SJ, Brown J, Bryksin A, Pinto A, Hatt JK, Konstantinidis KT (2023). Parallel deployment of passive and composite samplers for surveillance and variant profiling of SARS-CoV-2 in sewage. Sci Total Environ.

[CR21] Chavarria-Miro G, Anfruns-Estrada E, Martinez-Velazquez A, Vazquez-Portero M, Guix S, Paraira M, Galofre B, Sanchez G, Pinto RM, Bosch A (2021). Time evolution of severe acute respiratory syndrome coronavirus 2 (SARS-CoV-2) in wastewater during the first pandemic wave of COVID-19 in the metropolitan area of Barcelona, Spain. Appl Environ Microbiol.

[CR22] Chen J, Wang R, Gilby NB, Wei G-W (2022). Omicron variant (B.1.1.529): infectivity, vaccine breakthrough, and antibody resistance. J Chem Inf Model.

[CR23] Claro ICM, Cabral AD, Augusto MR, Duran AFA, Graciosa MCP, Fonseca FLA, Speranca MA, de Bueno RF (2021). Long-term monitoring of SARS-COV-2 RNA in wastewater in Brazil: a more responsive and economical approach. Water Res.

[CR24] Cleveland WS (1979). Robust locally weighted regression and smoothing scatterplots. J Am Stat Assoc.

[CR25] Coccia M (2020). Factors determining the diffusion of COVID-19 and suggested strategy to prevent future accelerated viral infectivity similar to COVID. Sci Total Environ.

[CR26] Coccia M (2021). Pandemic prevention: lessons from COVID-19. Encyclopedia.

[CR27] Coccia M (2022). Optimal levels of vaccination to reduce COVID-19 infected individuals and deaths: a global analysis. Environ Res.

[CR28] Coccia M (2022). Preparedness of countries to face COVID-19 pandemic crisis: strategic positioning and factors supporting effective strategies of prevention of pandemic threats. Environ Res.

[CR29] Daleiden B, Niederstätter H, Steinlechner M, Wildt S, Kaiser M, Lass-Flörl C, Posch W, Fuchs S, Pfeifer B, Huber A, Oberacher H (2022). Wastewater surveillance of SARS-CoV-2 in Austria: development, implementation, and operation of the Tyrolean wastewater monitoring program. J Water Health.

[CR30] Danecek P, Bonfield JK, Liddle J, Marshall J, Ohan V, Pollard MO, Whitwham A, Keane T, McCarthy SA, Davies RM, Li H (2021). Twelve years of SAMtools and BCFtools. Gigascience.

[CR31] Duintjer Tebbens RJ, Zimmermann M, Pallansch MA, Thompson KM (2017). Insights from a systematic search for information on designs, costs, and effectiveness of poliovirus environmental surveillance systems. Food Environ Virol.

[CR32] EC (2021a) Commission Recommendation (EU) 2021a/472 of 17 March 2021a on a common approach to establish a systematic surveillance of SARS-CoV-2 and its variants in wastewaters in the EU. EUR-Lex, an official website of the European Union. https://eur-lex.europa.eu/eli/reco/2021/472/oj Accessed 20 December 2021

[CR33] EC (2021b) Council Recommendation (EU) 2021b/132 of 2 February 2021b amending Recommendation (EU) 2020/912 on the temporary restriction on non-essential travel into the EU and the possible lifting of such restriction. EUR-Lex, an official website of the European Union. https://eur-lex.europa.eu/eli/reco/2021/132/oj. Accessed 20 November 2021

[CR34] ECDC (2020) SARS-CoV-2 variants of concern as of 25 December 2020. European Centre for Disease Prevention and Control. https://www.ecdc.europa.eu/en/covid-19/variants-concern. Accessed 25 December 2020

[CR35] Fan J, Gijbels I (1996) Local Polynomial Modelling and Its Applications: Monographs on Statistics and Applied Probability, vol 66, 1st edn. Routledge. 10.1201/9780203748725

[CR36] Fontenele RS, Kraberger S, Hadfield J, Driver EM, Bowes D, Holland LA, Faleye TOC, Adhikari S, Kumar R, Inchausti R, Holmes WK, Deitrick S, Brown P, Duty D, Smith T, Bhatnagar A, Yeager RA, Holm RH, von Reitzenstein NH, Wheeler E, Dixon K, Constantine T, Wilson MA, Lim ES, Jiang X, Halden RU, Scotch M, Varsani A (2021). High-throughput sequencing of SARS-CoV-2 in wastewater provides insights into circulating variants. Water Res.

[CR37] Francisco-Fernández M, Tarrío-Saavedra J, Naya S, López-Beceiro J, Artiaga R (2015). Classification of wood using differential thermogravimetric analysis. J Therm Anal Calorim.

[CR38] Gafurov A, Baláž A, Amman F, Boršová K, Čabanová V, Klempa B, Bergthaler A, Vinař T, Brejová B (2022) VirPool: model-based estimation of SARS-CoV-2 variant proportions in wastewater samples. medRxiv 2022.06.21.22276717. 10.1101/2022.06.21.22276717 10.1186/s12859-022-05100-3 PMC9761630 36536300

[CR39] Galani A, Aalizadeh R, Kostakis M, Markou A, Alygizakis N, Lytras T, Adamopoulos PG, Peccia J, Thompson DC, Kontou A, Karagiannidis A, Lianidou ES, Avgeris M, Paraskevis D, Tsiodras S, Scorilas A, Vasiliou V, Dimopoulos MA, Thomaidis NS (2022). SARS-CoV-2 wastewater surveillance data can predict hospitalizations and ICU admissions. Sci Total Environ.

[CR40] Gasser T, Kneip A, Köhler W (1991). A flexible and fast method for automatic smoothing. J Am Stat Assoc.

[CR41] Gerrity D, Papp K, Stoker M, Sims A, Frehner W (2021). Early-pandemic wastewater surveillance of SARS-CoV-2 in Southern Nevada: methodology, occurrence, and incidence/prevalence considerations. Water Res X.

[CR42] Girón-Guzmán I, Díaz-Reolid A, Cuevas-Ferrando E, Falcó I, Cano-Jiménez P, Comas I, Pérez-Cataluña A, Sánchez G (2023). Evaluation of two different concentration methods for surveillance of human viruses in sewage and their effects on SARS-CoV-2 sequencing. Sci Total Environ.

[CR43] Gobeil SM-C, Janowska K, McDowell S, Mansouri K, Parks R, Stalls V, Kopp MF, Manne K, Li D, Wiehe K, Saunders KO, Edwards RJ, Korber B, Haynes BF, Henderson R, Acharya P (2021). Effect of natural mutations of SARS-CoV-2 on spike structure, conformation, and antigenicity. Science.

[CR44] Grubaugh ND, Gangavarapu K, Quick J, Matteson NL, De Jesus JG, Main BJ, Tan AL, Paul LM, Brackney DE, Grewal S, Gurfield N, Van Rompay KKA, Isern S, Michael SF, Coffey LL, Loman NJ, Andersen KG (2019). An amplicon-based sequencing framework for accurately measuring intrahost virus diversity using PrimalSeq and iVar. Genome Biol.

[CR45] Guo M, Tao W, Flavell RA, Zhu S (2021). Potential intestinal infection and faecal-oral transmission of SARS-CoV-2. Nat Rev Gastroenterol Hepatol.

[CR46] Harvey WT, Carabelli AM, Jackson B, Gupta RK, Thomson EC, Harrison EM, Ludden C, Reeve R, Rambaut A, Peacock SJ, Robertson DL (2021). SARS-CoV-2 variants, spike mutations and immune escape. Nat Rev Microbiol.

[CR47] Hellmér M, Paxéus N, Magnius L, Enache L, Arnholm B, Johansson A, Bergström T, Norder H (2014) Detection of pathogenic viruses in wastewater provided early warnings of hepatitis A virus and norovirus outbreaks. Appl Environ Microbiol 80:6771–6781. 10.1128/AEM.01981-14 PMC4249052 25172863

[CR48] Herrmann E (1997). Local bandwidth choice in kernel regression estimation. J Comput Graph Stat.

[CR49] Herrmann E, Maechler M (2021) lokern: kernel regression smoothing with local or global plug-in bandwidth. R package version 1.1–9. CRAN. https://cran.r-project.org/web/packages/lokern/index.html Accessed 20 June 2020

[CR50] Hu Y, Shen L, Xu Z, Zhou J, Zhou H (2020) SARS-CoV-2 may persist in digestive tract longer than respiratory tract. Preprints 2020020354. 10.20944/preprints202002.0354.v1

[CR51] INE (2019) Data by census sections at 1 January 2019. INEbase. https://www.ine.es/jaxi/tabla.do?path=/t20/e245/p07/a2019/l1/&file=1502.px&type=pcaxis&L=1 Accessed 1 July 2020

[CR52] Jahn K, Dreifuss D, Topolsky I, Kull A, Ganesanandamoorthy P, Fernandez-Cassi X, Bänziger C, Devaux AJ, Stachler E, Caduff L, Cariti F, Corzón AT, Fuhrmann L, Chen C, Jablonski KP, Nadeau S, Feldkamp M, Beisel C, Aquino C, Stadler T, Ort C, Kohn T, Julian TR, Beerenwinkel N (2022). Early detection and surveillance of SARS-CoV-2 genomic variants in wastewater using COJAC. Nat Microbiol.

[CR53] Jarvie MM, Reed-Lukomski M, Southwell B, Wright D, Nguyen TNT (2023). Monitoring of COVID-19 in wastewater across the Eastern Upper Peninsula of Michigan. Environ Adv.

[CR54] Jiang X, Luo M, Zou Z, Wang X, Chen C, Qiu J (2020). Asymptomatic SARS-CoV-2 infected case with viral detection positive in stool but negative in nasopharyngeal samples lasts for 42 days. J Med Virol.

[CR55] Johnson R, Sharma JR, Ramharack P, Mangwana N, Kinnear C, Viraragavan A, Glanzmann B, Louw J, Abdelatif N, Reddy T, Surujlal-Naicker S, Nkambule S, Mahlangeni N, Webster C, Mdhluli M, Gray G, Mathee A, Preiser W, Muller C, Street R (2022) Tracking the circulating SARS-CoV-2 variant of concern in South Africa using wastewater-based epidemiology. Sci Rep 12:1182. 10.1038/s41598-022-05110-4 10.1038/s41598-022-05110-4 PMC8783013 35064174

[CR56] Joshi M, Kumar M, Srivastava V, Kumar D, Rathore DS, Pandit R, Graham DW, Joshi CG (2022). Genetic sequencing detected the SARS-CoV-2 delta variant in wastewater a month prior to the first COVID-19 case in Ahmedabad (India). Environ Pollut.

[CR57] Karthikeyan S, Levy JI, De Hoff P, Humphrey G, Birmingham A, Jepsen K, Farmer S, Tubb HM, Valles T, Tribelhorn CE, Tsai R, Aigner S, Sathe S, Moshiri N, Henson B, Mark AM, Hakim A, Baer NA, Barber T, Belda-Ferre P, Chacón M, Cheung W, Cresini ES, Eisner ER, Lastrella AL, Lawrence ES (2022). Wastewater sequencing reveals early cryptic SARS-CoV-2 variant transmission. Nature.

[CR58] Kirby AE, Welsh RM, Marsh ZA, Yu AT, Vugia DJ, Boehm AB, Wolfe MK, White BJ, Matzinger SR, Wheeler A, Bankers L, Andresen K, Salatas C, Gregory DA, Johnson MC, Trujillo M, Kannoly S, Smyth DS, Dennehy JJ, Sapoval N, Ensor K, Treangen T, Stadler LB, Hopkins L, New York City Department of Environmental Protection (2022). Notes from the field: early evidence of the SARS-CoV-2 B.1.1.529 (Omicron) variant in community wastewater — United States, November–December 2021. MMWR Morb Mortal Wkly Rep.

[CR59] Kolarević S, Micsinai A, Szántó-Egész R, Lukács A, Kračun-Kolarević M, Djordjevic A, Vojnović-Milutinović D, Marić JJ, Kirschner AKT, Farnleitner AAH, Linke R, Đukic A, Kostić-Vuković J, Paunović M (2022). Wastewater-based epidemiology in countries with poor wastewater treatment — epidemiological indicator function of SARS-CoV-2 RNA in surface waters. Sci Total Environ.

[CR60] Kopel E, Kaliner E, Grotto I (2014). Lessons from a public health emergency — importation of wild poliovirus to Israel. N Eng J Med.

[CR61] Kuhn KG, Jarshaw J, Jeffries E, Adesigbin K, Maytubby P, Dundas N, Miller AC, Rhodes E, Stevenson B, Vogel J, Reeves H (2022). Predicting COVID-19 cases in diverse population groups using SARS-CoV-2 wastewater monitoring across Oklahoma City. Sci Total Environ.

[CR62] Kumar M, Joshi M, Shah AV, Srivastava V, Dave S (2021). Wastewater surveillance-based city zonation for effective COVID-19 pandemic preparedness powered by early warning: a perspectives of temporal variations in SARS-CoV-2-RNA in Ahmedabad, India. Sci Total Environ.

[CR63] Kumar S, Thambiraja TS, Karuppanan K, Subramaniam G (2022). Omicron and Delta variant of SARS-CoV-2: a comparative computational study of spike protein. J Med Virol.

[CR64] Kumblathan T, Liu Y, Qiu Y, Pang L, Hrudey SE, Le XC, Li X-F (2023). An efficient method to enhance recovery and detection of SARS-CoV-2 RNA in wastewater. J Environ Sci.

[CR65] Lai CC, Liu YH, Wang CY, Wang YH, Hsueh SC, Yen MY, Ko WC, Hsueh PR (2020). Asymptomatic carrier state, acute respiratory disease, and pneumonia due to severe acute respiratory syndrome coronavirus 2 (SARS-CoV-2): Facts and myths. J Microbiol Immunol Infect.

[CR66] Lan J, Ge J, Yu J, Shan S, Zhou H, Fan S, Zhang Q, Shi X, Wang Q, Zhang L, Wang X (2020). Structure of the SARS-CoV-2 spike receptor-binding domain bound to the ACE2 receptor. Nature.

[CR67] Langeveld J, Schilperoort R, Heijnen L, Elsinga G, Schapendonk CEM, Fanoy E, de Schepper EIT, Koopmans MPG, de Graaf M, Medema G (2023). Normalisation of SARS-CoV-2 concentrations in wastewater: The use of flow, electrical conductivity and crAssphage. Sci Total Environ.

[CR68] Li L, Mazurowski L, Dewan A, Carine M, Haak L, Guarin TC, Dastjerdi NG, Gerrity D, Mentzer C, Pagilla KR (2022). Longitudinal monitoring of SARS-CoV-2 in wastewater using viral genetic markers and the estimation of unconfirmed COVID-19 cases. Sci Total Environ.

[CR69] Li H (2013) Aligning sequence reads, clone sequences and assembly contigs with BWA-MEM. arXiv:1303.3997. 10.48550/arXiv.1303.3997

[CR70] López de Ullibarri I, Tomás L, Freire B, Vaamonde M, Gallego, P, Barbeito I, Trigo-Tasende N, Vallejo JA, Tarrío-Saavedra J, Alvariño P, Beade E, Estévez N, Rumbo-Feal S, de Chiara L, Iglesias I, Poza M, Ladra S, Posada D, Cao R (2023) SARS-CoV-2 variant prevalence estimation using wastewater samples. medRxiv. 10.1101/2023.01.13.23284507

[CR71] Lyngse FP, Mølbak K, Skov RL, Christiansen LE, Mortensen LH, Albertsen M, Møller CH, Krause TG, Rasmussen M, Michaelsen TY, Voldstedlund M, Fonager J, Steenhard N, Kirkeby CT (2021). Increased transmissibility of SARS-CoV-2 lineage B.1.1.7 by age and viral load. Nat Commun.

[CR72] Lyngse FP, Kirkeby CT, Denwood M, Christiansen LE, Mølbak K, Møller CH, Skov RL, Krause TG, Rasmussen M, Sieber RN, Johannesen TB, Lillebaek T, Fonager J, Fomsgaard A, Møller FT, Stegger M, Overvad M, Spiess K, Mortensen LH (2022). Household transmission of SARS-CoV-2 Omicron variant of concern subvariants BA.1 and BA.2 in Denmark. Nat Commun.

[CR73] Maida CM, Amodio E, Mazzucco W, La Rosa G, Lucentini L, Suffredini E, Palermo M, Andolina G, Iaia FR, Merlo F, Chiarelli MG, Siragusa A, Vitale F, Tramuto F, Segreto D, Schembri P, Cuffari G, Conti A, Casamassima G, Polizzi A, Ferrara M, Gullo G, Lo Verde A, Russo A, Casuccio A, Costantino C, Restivo V, Immordino P, Graziano G (2022). Wastewater-based epidemiology for early warning of SARS-COV-2 circulation: a pilot study conducted in Sicily, Italy. Int J Hyg Environ Health.

[CR74] Masachessi G, Castro G, Cachi AM, de los Marinzalda MÁ, Liendo M, Pisano MB, Sicilia P, Ibarra G, Rojas RM, López L, Barbás G, Cardozo D, Ré VE, Nates SV (2022). Wastewater based epidemiology as a silent sentinel of the trend of SARS-CoV-2 circulation in the community in central Argentina. Water Res.

[CR75] McMahan CS, Self S, Rennert L, Kalbaugh C, Kriebel D, Graves D, Colby C, Deaver JA, Popat SC, Karanfil T, Freedman DL (2021). COVID-19 wastewater epidemiology: a model to estimate infected populations. Lancet Planet Health.

[CR76] Migueres M, Dimeglio C, Trémeaux P, Abravanel F, Raymond S, Lhomme S, Mansuy J-M, Izopet J (2022). Influence of immune escape and nasopharyngeal virus load on the spread of SARS-CoV-2 Omicron variant. J Infect.

[CR77] Monteiro S, Rente D, Cunha MV, Gomes MC, Marques TA, Lourenco AB, Cardoso E, Alvaro P, Silva M, Coelho N, Vilaca J, Meireles F, Broco N, Carvalho M, Santos R (2022). A wastewater-based epidemiology tool for COVID-19 surveillance in Portugal. Sci Total Environ.

[CR78] NORMAN (2021) NORMAN SCORE Database - SARS-CoV-2 in sewage (SC2S). NORMAN Database System. https://www.norman-network.com/nds/sars_cov_2/. Accessed 3 September 2021

[CR79] Núñez-Delgado A, Bontempi E, Coccia M, Kumar M, Farkas K, Domingo JL (2021). SARS-CoV-2 and other pathogenic microorganisms in the environment. Environ Res.

[CR80] Padilla-Reyes DA, Álvarez MM, Mora A, Cervantes-Avilés PA, Kumar M, Loge FJ, Mahlknecht J (2022). Acquired insights from the long-term surveillance of SARS-CoV-2 RNA for COVID-19 monitoring: the case of Monterrey Metropolitan Area (Mexico). Environ Res.

[CR81] Parums D (2022). Editorial: World Health Organization (WHO) Variants of concern lineages under monitoring (VOC-LUM) in response to the global spread of lineages and sublineages of omicron, or B.1.1.529, SARS-CoV-2. Med Sci Monit.

[CR82] Pechlivanis N, Tsagiopoulou M, Maniou MC, Togkousidis A, Mouchtaropoulou E, Chassalevris T, Chaintoutis SC, Petala M, Kostoglou M, Karapantsios T, Laidou S, Vlachonikola E, Chatzidimitriou A, Papadopoulos A, Papaioannou N, Dovas CI, Argiriou A, Psomopoulos F (2022). Detecting SARS-CoV-2 lineages and mutational load in municipal wastewater and a use-case in the metropolitan area of Thessaloniki. Greece Sci Rep.

[CR83] Pérez-Cataluña A, Chiner-Oms Á, Cuevas-Ferrando E, Díaz-Reolid A, Falcó I, Randazzo W, Girón-Guzmán I, Allende A, Bracho MA, Comas I, Sánchez G (2022). Spatial and temporal distribution of SARS-CoV-2 diversity circulating in wastewater. Water Res.

[CR84] Peterson SW, Lidder R, Daigle J, Wonitowy Q, Dueck C, Nagasawa A, Mulvey MR, Mangat CS (2022). RT-qPCR detection of SARS-CoV-2 mutations S 69–70 del, S N501Y and N D3L associated with variants of concern in Canadian wastewater samples. Sci Total Environ.

[CR85] Pillay L, Amoah ID, Deepnarain N, Pillay K, Awolusi OO, Kumari S, Bux F (2021). Monitoring changes in COVID-19 infection using wastewater-based epidemiology: a South African perspective. Sci Total Environ.

[CR86] Pipes L, Chen Z, Afanaseva S, Nielsen R (2022) Estimating the relative proportions of SARS-CoV-2 strains from wastewater samples. medRxiv. 2022.01.13.22269236. 10.1101/2022.01.13.22269236 10.1016/j.crmeth.2022.100313 PMC9485417 36159190

[CR87] Prado T, Rey-Benito G, Miagostovich MP, Sato MIZ, Rajal VB, Filho CRM, Pereira AD, Barbosa MRF, Mannarino CF, da Silva AS (2023). Wastewater-based epidemiology for preventing outbreaks and epidemics in Latin America – lessons from the past and a look to the future. Sci Total Environ.

[CR88] R Core Team (2022) R: A language and environment for statistical computing. R Foundation for Statistical Computing, Vienna, Austria. http://www.r-project.org/index.html. Accessed 20 June 2020

[CR89] Rafiee M, Isazadeh S, Mohseni-Bandpei A, Mohebbi SR, Jahangiri-rad M, Eslami A, Dabiri H, Roostaei K, Tanhaei M, Amereh F (2021). Moore swab performs equal to composite and outperforms grab sampling for SARS-CoV-2 monitoring in wastewater. Sci Total Environ.

[CR90] Reynolds LJ, Gonzalez G, Sala-Comorera L, Martin NA, Byrne A, Fennema S, Holohan N, Kuntamukkula SR, Sarwar N, Nolan TM, Stephens JH, Whitty M, Bennett C, Luu Q, Morley U, Yandle Z, Dean J, Joyce E, O’Sullivan JJ, Cuddihy JM, McIntyre AM, Robinson EP, Dahly D, Fletcher NF, Carr M, De Gascun C, Meijer WG (2022). SARS-CoV-2 variant trends in Ireland: wastewater-based epidemiology and clinical surveillance. Sci Total Environ.

[CR91] Riediker M, Briceno-Ayala L, Ichihara G, Albani D, Poffet D, Tsai D-H, Iff S, Monn C (2022). Higher viral load and infectivity increase risk of aerosol transmission for Delta and Omicron variants of SARS-CoV-2. Swiss Med Wkly.

[CR92] Riou C, Keeton R, Moyo-Gwete T, Hermanus T, Kgagudi P, Baguma R, Valley-Omar Z, Smith M, Tegally H, Doolabh D, Iranzadeh A, Tyers L, Mutavhatsindi H, Tincho MB, Benede N, Marais G, Chinhoyi LR, Mennen M, Skelem S, du Bruyn E, Stek C, de Oliveira T, Williamson C, Moore PL, Wilkinson RJ, Ntusi NAB, Burgers WA, South African cellular immunity network (2022). Escape from recognition of SARS-CoV-2 variant spike epitopes but overall preservation of T cell immunity. Sci Transl Med.

[CR93] Robotto A, Lembo D, Quaglino P, Brizio E, Polato D, Civra A, Cusato J, Di Perri G (2022). Wastewater-based SARS-CoV-2 environmental monitoring for Piedmont, Italy. Environ Res.

[CR94] Rothe C, Schunk M, Sothmann P, Bretzel G, Froeschl G, Wallrauch C, Zimmer T, Thiel V, Janke C, Guggemos W, Seilmaier M, Drosten C, Vollmar P, Zwirglmaier K, Zange S, Wölfel R, Hoelscher M (2020). Transmission of 2019-nCoV infection from an asymptomatic contact in Germany. N Engl J Med.

[CR95] Rubio-Acero R, Beyerl J, Muenchhoff M, Roth MS, Castelletti N, Paunovic I, Radon K, Springer B, Nagel C, Boehm B, Böhmer MM, Graf A, Blum H, Krebs S, Keppler OT, Osterman A, Khan ZN, Hoelscher M, Wieser A (2021). Spatially resolved qualified sewage spot sampling to track SARS-CoV-2 dynamics in Munich - one year of experience. Sci Total Environ.

[CR96] Saingam P, Li B, Nguyen Quoc B, Jain T, Bryan A, Winkler MKH (2023). Wastewater surveillance of SARS-CoV-2 at intra-city level demonstrated high resolution in tracking COVID-19 and calibration using chemical indicators. Sci Total Environ.

[CR97] Sangsanont J, Rattanakul S, Kongprajug A, Chyerochana N, Sresung M, Sriporatana N, Wanlapakorn N, Poovorawan Y, Mongkolsuk S, Sirikanchana K (2022). SARS-CoV-2 RNA surveillance in large to small centralized wastewater treatment plants preceding the third COVID-19 resurgence in Bangkok, Thailand. Sci Total Environ.

[CR98] Sapoval, N, Lou E, Hopkins L, Ensor KB, Schneider R, Treangen TJ, Stadler LB (2021) Enhanced detection of recently emerged SARS-CoV-2 variants of concern in wastewater. medRxiv. 2021.09.08.21263279. 10.1101/2021.09.08.21263279 10.1038/s41467-023-38184-3 PMC10191095 37198181

[CR99] Saththasivam J, El-Malah SS, Gomez TA, Jabbar KA, Remanan R, Krishnankutty AK, Ogunbiyi O, Rasool K, Ashhab S, Rashkeev S, Bensaad M, Ahmed AA, Mohamoud YA, Malek JA, Abu Raddad LJ, Jeremijenko A, Abu Halaweh HA, Lawler J, Mahmoud KA (2021). COVID-19 (SARS-CoV-2) outbreak monitoring using wastewater-based epidemiology in Qatar. Sci Total Environ.

[CR100] Sentis C, Billaud G, Bal A, Frobert E, Bouscambert M, Destras G, Josset L, Lina B, Morfin F, Gaymard, the COVID-Diagnosis HCL Study Group (2022). SARS-CoV-2 omicron variant, lineage BA.1, is associated with lower viral load in nasopharyngeal samples compared to delta variant. Viruses.

[CR101] Tanimoto Y, Ito E, Miyamoto S, Mori A, Nomoto R, Nakanishi N, Oka N, Morimoto T, Iwamoto T (2022). SARS-CoV-2 RNA in wastewater was highly correlated with the number of COVID-19 cases during the fourth and fifth pandemic wave in Kobe City, Japan. Front Microbiol.

[CR102] Tharak A, Kopperi H, Hemalatha M, Kiran U, Gokulan CG, Moharir S, Mishra RK, Mohan SV (2022). Longitudinal and long-term wastewater surveillance for COVID-19: infection dynamics and zoning of urban community. Int J Environ Res Public Health.

[CR103] Tian D, Sun Y, Zhou J, Ye Q (2021). The global epidemic of the SARS-CoV-2 delta variant, key spike mutations and immune escape. Front Immunol.

[CR104] Valieris R, Drummond RD, Defelicibus A, Dias-Neto E, Rosales RA, Tojal da Silva I (2022) A mixture model for determining SARS-Cov-2 variant composition in pooled samples. Bioinformatics btac047. 10.1093/bioinformatics/btac047 10.1093/bioinformatics/btac047 35104309

[CR105] Vallejo JA, Trigo-Tasende N, Rumbo-Feal S, Conde-Pérez K, López-Oriona Á, Barbeito I, Vaamonde M, Tarrío-Saavedra J, Reif R, Ladra S, Rodiño-Janeiro BK, Nasser-Ali M, Cid Á, Veiga M, Acevedo A, Lamora C, Bou G, Cao R, Poza M (2022). Modeling the number of people infected with SARS-COV-2 from wastewater viral load in Northwest Spain. Sci Total Environ.

[CR106] Vellas C, Delobel P, De Souto BP, Izopet J (2020). COVID-19, virology and geroscience: a perspective. J Nutr Health Aging.

[CR107] Vieu P (1991). Nonparametric regression: optimal local bandwidth choice. J R Stat Soc Series B Stat Methodol.

[CR108] Vo V, Tillett RL, Papp K, Shen S, Gu R, Gorzalski A, Siao D, Markland R, Chang C-L, Baker H, Chen J, Schiller M, Betancourt WQ, Buttery E, Pandori M, Picker MA, Gerrity D, Oh EC (2022). Use of wastewater surveillance for early detection of Alpha and Epsilon SARS-CoV-2 variants of concern and estimation of overall COVID-19 infection burden. Sci Total Environ.

[CR109] Wand MP, Jones MC (1994) Kernel Smoothing, 1st edn. Chapman and Hall/CRC. https://doi.org/10.1201/b14876

[CR110] Wand M (2021) KernSmooth: Functions for Kernel Smoothing Supporting Wand & Jones (1995). R package version 2.23–20. CRAN. https://CRAN.R-project.org/package=KernSmooth. Accessed 20 June 2020

[CR111] Weidhaas J, Aanderud ZT, Roper DK, VanDerslice J, Gaddis EB, Ostermiller J, Hoffman K, Jamal R, Heck P, Zhang Y, Torgersen K, Laan JV, LaCross N (2021). Correlation of SARS-CoV-2 RNA in wastewater with COVID-19 disease burden in sewersheds. Sci Total Environ.

[CR112] WHO (2020) WHO Director-General’s opening remarks at the media briefing on COVID-19 - 23 October 2020. World Health Organization. https://www.who.int/director-general/speeches/detail/who-director-general-s-opening-remarks-at-the-media-briefing-on-covid-19---23-october-2020. Accessed 23 October 2020

[CR113] Wilder ML, Middleton F, Larsen DA, Du Q, Fenty A, Zeng T, Insaf T, Kilaru P, Collins M, Kmush B, Green HC (2021). Co-quantification of crAssphage increases confidence in wastewater-based epidemiology for SARS-CoV-2 in low prevalence areas. Water Res X.

[CR114] Wood SN (2017) Generalized Additive Models: An Introduction with R, Second Edition, 2nd ed. Chapman and Hall/CRC. 10.1201/9781315370279

[CR115] Wu F, Zhang J, Xiao A, Gu X, Lee WL, Armas F, Kauffman K, Hanage W, Matus M, Ghaeli N, Endo N, Duvallet C, Poyet M, Moniz K, Washburne AD, Erickson TB, Chai PR, Thompson J, Alm EJ (2020). SARS-CoV-2 titers in wastewater are higher than expected from clinically confirmed cases. mSystems.

[CR116] Wu F, Xiao A, Zhang J, Moniz K, Endo N, Armas F, Bonneau R, Brown MA, Bushman M, Chai PR, Duvallet C, Erickson TB, Foppe K, Ghaeli N, Gu X, Hanage WP, Huang KH, Lee WL, Matus M, McElroy KA, Nagler J, Rhode SF, Santillana M, Tucker JA, Wuertz S, Zhao S, Thompson J, Alm EJ (2022). SARS-CoV-2 RNA concentrations in wastewater foreshadow dynamics and clinical presentation of new COVID-19 cases. Sci Total Environ.

[CR117] Xiao F, Tang M, Zheng X, Liu Y, Li X, Shan H (2020). Evidence for gastrointestinal infection of SARS-CoV-2. Gastroenterology.

[CR118] Yanaç K, Adegoke A, Wang L, Uyaguari M, Yuan Q (2022) Detection of SARS-CoV-2 RNA throughout wastewater treatment plants and a modeling approach to understand COVID-19 infection dynamics in Winnipeg, Canada. Sci Total Environ 825:153906. 10.1016/j.scitotenv.2022.153906 10.1016/j.scitotenv.2022.153906 PMC8864809 35218826

[CR119] Yaniv K, Shagan M, Lewis YE, Kramarsky-Winter E, Weil M, Indenbaum V, Elul M, Erster O, Brown AS, Mendelson E, Mannasse B, Shirazi R, Lakkakula S, Miron O, Rinott E, Baibich RG, Bigler I, Malul M, Rishti R, Brenner A, Friedler E, Gilboa Y, Sabach S, Alfiya Y, Cheruti U, Davidovich N, Moran-Gilad J, Berchenko Y, Bar-Or I, Kushmaro A (2021). City-level SARS-CoV-2 sewage surveillance. Chemosphere.

[CR120] Zhang D, Ling H, Huang X, Li J, Li W, Yi C, Zhang T, Jiang Y, He Y, Deng S, Zhang X, Wang X, Liu Y, Li G, Qu J (2020). Potential spreading risks and disinfection challenges of medical wastewater by the presence of Severe Acute Respiratory Syndrome Coronavirus 2 (SARS-CoV-2) viral RNA in septic tanks of Fangcang Hospital. Sci Total Environ.

[CR121] Zhang X, Wu S, Wu B, Yang Q, Chen A, Li Y, Zhang Y, Pan T, Zhang H, He X (2021). SARS-CoV-2 Omicron strain exhibits potent capabilities for immune evasion and viral entrance. Signal Transduct Target Ther.

[CR122] Zhao L, Zou Y, David RE, Withington S, McFarlane S, Faust RA, Norton J, Xagoraraki I (2023). Simple methods for early warnings of COVID-19 surges: lessons learned from 21 months of wastewater and clinical data collection in Detroit, Michigan, United States. Sci Total Environ.

[CR123] Zheng S, Fan J, Yu F, Feng B, Lou B, Zou Q, Xie G, Lin S, Wang R, Yang X, Chen W, Wang Q, Zhang D, Liu Y, Gong R, Ma Z, Lu S, Xiao Y, Gu Y, Zhang J, Yao H, Xu K, Lu X, Wei G, Zhou J, Fang Q, Cai H, Qiu Y, Sheng J, Chen Y, Liang T (2020). Viral load dynamics and disease severity in patients infected with SARS-CoV-2 in Zhejiang province, China, January-March 2020: retrospective cohort study. BMJ.

