# Supplementary material for: Wastewater early warning system for SARS-CoV-2 outbreaks and variants in a Coruña, Spain
Source: Environ Sci Pollut Res Int. 2023 Jun 7;30(32):79315–34. doi: 10.1007/s11356-023-27877-3 (PMC10247271; doi:10.1007/s11356-023-27877-3)
Supplement: Supplementary file 1 — Supplementary file1 (DOCX 16 KB) [file 11356_2023_27877_MOESM1_ESM.docx]

**SUPPLEMENTARY MATERIAL (ESM_1)**
**Environmental Science and Pollution Research**

**COVIDBENS: SARS-CoV-2 WASTEWATER EARLY WARNING SYSTEM IN A CORUÑA, SPAIN**

Noelia Trigo-Tasende**^#^**^1^, Juan A. Vallejo**^#^**^1^, Soraya Rumbo-Feal^1^, Kelly Conde-Pérez^a1^, Manuel Vaamonde^2^, Ángel López-Oriona^2^, Inés Barbeito^2^, Mohammed Nasser-Ali^1^, Rubén Reif^3^, Bruno K. Rodiño-Janeiro^4^, Elisa Fernández-Álvarez^5^, Iago Iglesias-Corrás^5^, Borja Freire^5^, Javier Tarrío-Saavedra^2^, Laura Tomás^6,7^, Pilar Gallego-García^6,7^, David Posada^6,7,8^, Germán Bou^1^, Ignacio López-de-Ullibarri^2^, Ricardo Cao*^2^, Susana Ladra*^5,^ and Margarita Poza*^1^

^1^ Microbiology Research Group: meiGAbiome-Biomedical Research Institute (INIBIC)-Center for Advanced Research (CICA)-University of A Coruña (UDC)-CIBER of Infectious Diseases (CIBERINFEC), Servicio de Microbiología, 3ª planta, Edificio Sur, Hospital Universitario, As Xubias, 15006 A Coruña, Spain.

^2^ Research Group MODES, Research Center for Information and Communication Technologies (CITIC), University of A Coruña (UDC), Campus de Elviña, 15071 A Coruña, Spain.

^3^ Center for Research in Biological Chemistry and Molecular Materials (CiQUS), University of Santiago de Compostela (USC), 15782 Santiago de Compostela, Spain.

^4^ BFlow, University of Santiago de Compostela (USC) and Health Research Institute of Santiago de Compostela (IDIS), Campus Vida, 15706 Santiago de Compostela, A Coruña, Spain.

^5^ University of A Coruña (UDC), Research Center for Information and Communication Technologies (CITIC), Database Laboratory, Campus de Elviña, 15071 A Coruña, Spain.

^6^ CINBIO, Universidade de Vigo, 36310 Vigo, Spain.

^7^ Galicia Sur Health Research Institute (IIS Galicia Sur), SERGAS-UVIGO, 36312 Vigo, Spain.

^8^ Department of Biochemistry, Genetics, and Immunology, Universidade de Vigo, 36310 Vigo, Spain.

**^#^** Authors contributed equally.

*Authors for correspondence and contributed equally:

Margarita Poza ([margarita.poza.dominguez@sergas.es](mailto:margarita.poza.dominguez@sergas.es); telephone number: +34981176552); Susana Ladra ([susana.ladra@udc.es](mailto:susana.ladra@udc.es)) and Ricardo Cao ([ricardo.cao@udc.es](mailto:ricardo.cao@udc.es)).

**Table S1.** Lower limit of detection (LoD) and limit of quantification (LoQ) determination for SARS-CoV-2 RT-qPCR.

| **Copies/ µL** | **Cq mean (SD)** | **Detected/tested (%)** | **RSD (%)** |
| --- | --- | --- | --- |
| 500 | 29.37 (0.64) | 20/20 (100) | 2.19 |
| 100 | 31.43 (0.68) | 20/20 (100) | 2.17 |
| 50 | 32.84 (1.11) | 20/20 (100) | 3.38 |
| 10 | 35.01 (1.06) | 20/20 (100) | 3.04 |
| 5 | 35.50 (0.82) | 19/20 (95) | 2.32 |

^RSD: relative standard deviation; SD: standard deviation^
